# Saikosaponin D exhibits anti-leukemic activity by targeting FTO/m^6^A signaling

**DOI:** 10.7150/thno.55574

**Published:** 2021-03-31

**Authors:** Kaiju Sun, Yangyang Du, Yuzhu Hou, Mingyue Zhao, Jiajia Li, Yazhe Du, Lingxiao Zhang, Changbao Chen, Hongmei Yang, Fei Yan, Rui Su

**Affiliations:** 1 Jilin Ginseng Academy, Changchun University of Chinese Medicine, Changchun, 130017, China.; 2 State Key Laboratory of Inorganic Synthesis and Preparative Chemistry, International Joint Research Laboratory of Nano-Micro Architecture Chemistry (NMAC), International Research Center for Chemistry-Medicine Joint Innovation, College of Chemistry, Jilin University, 2699 Qianjin Street, Changchun 130012, China.; 3 Department of Obstetrics and Gynecology, First Hospital, Jilin University, 130021, P. R. China.; 4 Department of blood specialty, First Hospital, Jilin University, 130021, China.

**Keywords:** Saikosaponin D, leukemia, N^6^-methyladenosine, FTO

## Abstract

**Purpose:** The implementation of targeted therapies for acute myeloid leukemia (AML) has been challenging. Fat mass and obesity associated protein (FTO), an mRNA N^6^-methyladenosine (m^6^A) demethylase, functions as an oncogene that promotes leukemic oncogene-mediated cell transformation and leukemogenesis. Here, we investigated the role of Saikosaponin-d (SsD) in broad anti-proliferation effects in AML and evaluated the m^6^A demethylation activity by targeting FTO of SsD.

**Methods:** It was examined whether and how SsD regulates FTO/m^6^A signaling in AML. The pharmacologic activities and mechanisms of actions of SsD *in vitro*, in mice, primary patient cells, and tyrosine kinase inhibitors-resistant cells were determined.

**Results:** SsD showed a broadly-suppressed AML cell proliferation and promoted apoptosis and cell-cycle arrest both *in vitro* and *in vivo*. Mechanistically, SsD directly targeted FTO, thereby increasing global m^6^A RNA methylation, which in turn decreased the stability of downstream gene transcripts, leading to the suppression of relevant pathways. Importantly, SsD also overcame FTO/m^6^A-mediated leukemia resistance to tyrosine kinase inhibitors.

**Conclusion:** Our findings demonstrated that FTO-dependent m^6^A RNA methylation mediated the anti-leukemic actions of SsD, thereby opening a window to develop SsD as an epitranscriptome-base drug for leukemia therapy.

## Introduction

Acute myeloid leukemia (AML) is a malignant disorder of hematopoietic stem cells which is characterized by clonal expansion of abnormally differentiated blasts of myeloid lineage 1, 2. Regarding AML treatment, small molecule inhibitors against each mutant target are widely used in clinical practice 3. The first targeted therapy for AML was used in patients with rearrangement of the retinoic acid receptor 4. Recently, several small-molecule inhibitors have been developed. Midostaurin, a broad-spectrum FLT3 inhibitor, was approved for use in newly diagnosed AML patients with FLT3 mutations, in combination with standard-of-care chemotherapy 5. IDH2 inhibitor, enasidenib 6, and the IDH1 inhibitor, ivosidenib 7, respectively inhibit mutant IDH2 and IDH1. Mubritinib, a known ERBB2 inhibitor, has shown strong anti-leukemic effects both *in vitro* and *in vivo* 8. Venetoclax, a selective BCL-2 inhibitor, has received FDA approval for the treatment of AML 9. However, prolonged treatment with this inhibitor results in drug resistance 10. Even then, the clinical outcomes for AML patients remain unsatisfactory with high mortality rates and therefore, necessitate using the novel genetic and epigenetic information to develop improved and more-targeted therapies 11.

M^6^A modification is the methylation of the adenosine base at the nitrogen-6 position of mRNA 12, 13. It is the most prevalent internal chemical modification of mRNAs in eukaryotes 14. Accruing evidence shows that m^6^A RNA methylation has an outsize effect on RNA production/metabolism and participates in the pathogenesis of multiple diseases including cancers 15, 16. The m^6^A levels depend on the functional interplay among several proteins. The fat mass and obesity (FTO)-associated gene, which belongs to the Fe^2+^- and 2-oxoglutarate (2OG)-dependent AlkB dioxygenase family, predominantly catalyze m^6^A demethylation 17. FTO is involved in various disease processes and has also been shown to be upregulated in several subtypes of AML 18. The discovery of small-molecule FTO inhibitors enables temporal intervention of mRNA methylation. The natural product rhein, an FTO inhibitor that competitively binds to the FTO active site *in vitro*, generally increases the cellular m^6^A on mRNA 19. Meclofenamic acid (MA), an anti-inflammatory drug, is a selective inhibitor of FTO demethylation of m^6^A rather than demethylase ALKBH5. In addition, MA is another key gene that is sensitive to the function of the m^6^A modulator, and can give rise to a significant change in phenotype. Due to substrate binding competition, MA inhibits FTO both selectively and efficiently 20. Structurally, oncometabolite R-2-hydroxyglutarate (R-2HG) is close to α-ketoglutarate (α-KG) and competitively inhibits a series of Fe(II)/α-KG-dependent dioxygenases. In addition, R-2HG also inhibits FTO activity, thereby increasing global N^6^-methyladenosine (m^6^A) RNA modification in R-2HG-sensitive leukemia cells 21. Other FTO inhibitors, such as FB23 and FB23-2 were developed using a structure-based rational design based on MA. These FTO inhibitors directly bind to FTO and thereby selectively inhibit its m^6^A demethylase activity 17.

Triterpenoids are chemical compounds with 30 carbon atoms made up of six isoprene units (C_5_H_8_). These are natural compounds with a variety of biological activities, mainly with anti-cancer and anti-viral effects 22. Other than these, triterpenoid saponins have also been highlighted for several other bioactivities, including anti-inflammatory, anti-allergic, anti-cancer effects, treatment of leukemia, and anti-viral activities 23. However, the underlying molecular mechanisms of its action are still unclear. Saikosaponin-d (SsD) is a triterpenoid saponin compound that is extracted from Radix Bupleuri, the dried root from the plant *Bupleurum falcatum.* SsD has been reported to exert multiple biological effects, including anti-cancer activity. In liver cancer treatment, SsD functions by suppressing cyclooxygenase (COX)-2 via the intermediary p-STAT3/C/EBPβ signaling pathway 24. Furthermore, in hepatoma cells, SsD has shown to reverse hypoxia promoted effects by activating the sentrin/small ubiquitin-like modifier (SUMO)-specific protease 5 (SENP5), which leads to the inhibition of SUMO1 and Gli 25. SsD has also been reported as a novel SERCA inhibitor that promotes autophagic cell death in apoptosis-defective cells 26. Moreover, SsD has shown to suppress cell growth in triple-negative breast cancer by targeting β-catenin signaling 27. However, there is a lack of knowledge elucidating the effect of SsD in leukemia.

Here, we showed that SsD displayed broad and intrinsic anti-tumor activity in leukemia both *in vivo* and *in vitro* by targeting FTO and its downstream pathways. These effects were due to altered mRNA m^6^A modification in the target RNAs. Thus, our findings revealed a previously unrecognized link between FTO/m^6^A-modification signaling and elucidated the function of SsD in AML.

## Methods

### Cell culture

The cell culture for leukemia cells was performed as our previous reports 28. Human leukemia cell lines NB4, Kasumi-1, K562, U937, HL60, SKNO-1, MV4-11, MOLM13, MOLM14, and mouse C1498 cells were obtained from the American Type Culture Collection (Manassas, VA, USA). NB4, K562, U937, HL60, MV4-11, MOLM13, and MOLM14 cells were cultured in RPMI1640 medium containing 10% fetal bovine serum (FBS) (Life Technology, New York, USA). Kasumi-1 and SKNO-1 cells were cultured in RPMI1640 with 20% FBS. C1498 cells were maintained in Dulbecco's Modified Eagle's Medium (DMEM) with 10% FBS.

### Cell proliferation, cell cycle, and cell apoptosis assays

To study the effects of SsD on cell viability, cells were seeded in triplicate in 96-well plates at a concentration of 5,000-10,000 cells per well. Cell proliferation and viability were assessed using the Cell Counting Kit-8 (CCK8; Dojindo Molecular Technologies) as per manufacturer's instructions. For cell cycle analysis, the Cell Cycle and Apoptosis Analysis Kit (Beyotime, Shanghai, China) was used. Cells were washed three times with ice-cold PBS, and were fixed in 70% ethanol in PBS at -20 °C for 12 h. After fixation, cells were washed with ice-cold PBS and stained with 0.5 mL of propidium iodide (PI) staining buffer, containing 200 mg/mL RNase A and 50 µg/mL PI, at 37 °C for 30 min in the dark. Finally, cells were analyzed using flow cytometry. For cell apoptosis analysis, cells were centrifuged (4 °C, 1000 rpm, 3 min), washed with PBS, stained using the Annexin V-FITC Apoptosis Detection Kit (Beyotime), and analyzed by flow cytometry. Analyses were performed on the BD LSR flow cytometer (BD Biosciences, New Jersey, USA). All experiments were performed in triplicate.

### Colony-forming assays

Colony-forming assays were performed using the MethoCult® mixture (Stem Cell Technologies, Canada) as per the manufacturer's guidelines described in a previous report 28, 29. Briefly, cells treated with different concentrations of SsD were suspended in 0.3 mL of IMDM plus 2% FBS (Stem Cell Technologies), mixed with MethoCult® medium, and then seeded into 35-mm dishes. Colonies were counted after 9-14 days.

### RNA-seq

RNA-seq was performed according to our previous report 30. Briefly, total RNA samples were extracted from NB4 cells treated with SsD or control for 48 h using a miRNeasy mini kit (Qiagen, Germany). Libraries were constructed on an Illumina Hiseq system. Differential gene expression was analyzed following a standard Illumina sequence analysis pipeline. Subsequently, Gene Set Enrichment Analysis (GSEA) was used to analyze the enriched signaling pathways.

### Molecular docking

The three-dimensional structure of SsD (PubChem CID:119307) was obtained from the NCBI Pubchem Compound database (http://www.ncbi.nlm.nih.gov/pccompound) by ChemDraw software, and the crystal structure of FTO (PDB ID: 2HYU6AKW) was obtained from the RCSB Protein Data Bank (http:// www.rcsb.org/pdb). Molecular docking was performed using AutoDock tools (version 4.2.6) at the default setting, based on the Lamarckian Genetic Algorithm (Scripps Research Institute, La Jolla, CA, USA). Hydrogens were added and all H_2_O_2_ molecules were removed using the Protein Preparation Wizard in Maestro v9.2. Then, the structure was energy-minimized. The chemical structure of SsD was prepared using LigPrep v2.5, then AMSOL partial atomic charges were assigned. The program Glide v5.7 was used for ligand docking. Flexible Docking was performed with extra precision mode. The number of poses per ligand was set to 10 in post-docking minimization and the best 5 poses were output. The other parameters were kept as default. The molecular structures were generated using PyMOL 1.6.x. We processed the optimum structure of the complex with the Discovery Studio 4.0 Visualizer (BIOVIA, San Diego, CA, USA).

### Cellular thermal shift assay (CETSA)

CETSA was conducted as described previously 31. NB4 and Kas-1 cells were harvested and lysed in 50 mM Tris-HCl (pH 7.5), 150 mM NaCl, and 2 mM DTT. To the supernatant, 10 μM SsD or DMSO was added and incubated at 25 °C for 25 min. After heat denaturing for 5 min, samples were centrifuged (4 °C, 12000 g, 10 min), and the supernatants were analyzed by Western blot analysis. All experiments were performed in triplicate.

### Nuclear magnetic resonance (NMR) titration

NMR data was acquired using phosphate buffer (20 mM sodium phosphate (pH 7.4), 100 mM NaCl, 5% DMSO) on a Bruker Avance NEO-400 MHz spectrometer equipped with a cryogenically cooled probe (Bruker biospin, Germany) at 25 °C. Experimental samples contained 100 μM SsD along with FTO or BSA protein at 0 μM, 1 μM, and 2 μM, respectively.

### Quantitation of SsD in AML cells

NB4 and Kas-1 cells were treated with 1 μM SsD for 48 h, respectively. Using 0.1% trypan blue, viable cells were stained, counted, and harvested after three times washings with PBS. Cells were diluted to 100 μL with 50% (v/v) water/methanol and subjected to three times shock freeze-thaw cycles for cell lysis. Supernatants were collected for HPLC (Agilent Technologies, Santa Clara, CA, USA) analysis using a HC-C18 column (Agilent) at 30 °C, and the absorbance was detected at 203 nm. The mobile phase consisted of acetonitrile and 0.1% phosphoric acid water and the flow rate was set to 1.0 mL/min.

### HPLC-based assay to measure the inhibition of m^6^A demethylation

Based on previously-published HPLC-based protocol 32, the reactions were performed in 50 mM Tris-HCl (pH 7.5-8.0) that contained 0.25 μM FTO_△N31_, 5 μM 15-mer ssRNA (5'-AUUGUCA(m^6^A)CAGCAGC-3'), 300 μM 2OG, 280 μM (NH_4_)_2_Fe(SO_4_)_2_, 2 mM L-ascorbic acid, and corresponding inhibitors at the indicated concentrations. Reactions were incubated at 25 °C for 30 min and terminated by heat denaturation at 90 °C for 5 min. Furthermore, the reaction mixtures were subjected to digestion with nuclease P1 (N8630, Sigma-aldrich) and alkaline phosphatase (P5931, Sigma). The half-maximal inhibitory concentration (IC_50_) values were obtained based on the inhibitory percentages of m^6^A demethylation for the corresponding inhibitor at the indicated concentrations, using nonlinear regression and dose-response fit in GraphPad Prism 5.0^TM^ software. All reactions were performed in triplicate.

### Detection of FTO enzymatic activity in a cell-free system using dot blot assays

The FTO demethylation activity was measured following a dot blot assay described previously 33. Recombinant human FTO protein was obtained from Abcam (ab109039). Single-stranded RNA with m^6^A modification, having the sequence 5'-AUUGUCA(m^6^A)CAGCAGC-3', was synthesized by Sangon Biotech (Shanghai). Demethylation activity assays were conducted in a 20 μL reaction mixture containing the indicated concentration of octyl ester modified SsD, 0.1 nmol ssRNA, 200 nM FTO, 283 μM of (NH_4_)_2_(SO_4_)_2_•6H_2_O (203505, Sigma-Aldrich), 75 μM of a-KG (K1128, Sigma-Aldrich), 2 mM of L-ascorbic acid (A0278, Sigma-Aldrich), 50 μg/mL of BSA (A2058, Sigma-Aldrich), and 50 mM of HEPES buffer, pH 7.0. The reactions were incubated at 37 °C for 3 h and quenched by the addition of 5 mM EDTA. This was followed by inactivation of the FTO enzyme by heating at 95 °C for 5 min. The ssRNAs were precipitated using one-tenth of a volume of 3 M sodium acetate (pH 5.2), glycogen (500 μg/mL, final concentration), and 2.5 volumes of 100% ethanol, and overnight incubation at -80°C. The RNA pellet was resuspended in 10 μL of RNase-free water and directly subjected to an m^6^A dot blot assay to determine m^6^A levels.

### RNA extraction and quantitative RT-PCR analysis

Total RNA was extracted using a miRNAeasy Kit (217004, Qiagen, Germany) and transcribed into cDNA by SuperScript III First-Strand Synthesis System (18080-051, Invitrogen, Canada) as per the manufacturer's guidelines 28. The qRT-PCR was performed with SYBR-Green (4309155) master mix on an ABI Prism 7000 sequence detection system (Applied Biosystems, Thermo Fisher Scientific). The expression of target genes, including *MYC, RARA,* and* ASB2* was normalized to 18 S expression levels. Relative gene expression levels were quantified using the 2^-ΔΔCT^ method. Gene-specific primer sequences are listed in Table S7.

### HPLC-MS/MS quantitation of m^6^A in AML cells

NB4 and Kas-1 cells were cultured with DMSO or SsD for 48 h, then mRNA was extracted and purified by the GenElute™ mRNA Miniprep Kit (Sigma #MRN70) as per the manufacturer's guidelines. A total of 1 μg of mRNA was digested using 2 U nuclease P1 (Sigma) in 40 μL buffer (10 mM Tris-HCl pH 7.0, 100 mM NaCl, 2.5 mM ZnCl_2_) at 37 °C for 12 h, followed by incubation with 1 U alkaline phosphatase at 37 °C for 2 h. The RNA solution was diluted 10-fold and 10 μL of the solution was injected into the system. Cellular levels of A, m^6^A were quantified using the HPLC (Ultimate 3000 system) coupled with a TSQ Quantiva mass spectrometer (Thermo, USA). Samples were centrifuged and loaded onto a Syncronis aQ C18 column (100 mm×2.1 mm, 1.7 μm, Thermo Scientific, USA) and eluted by the gradient methanol. The parent-to-product ions for quantification of A and m^6^A were at *m/z* 268.1/136.1 (A) and 282.1/150.1 (m^6^A), respectively.

### Plasmid and shRNA transfection

Human FTO (GeneID: 79068) gene sequences were cloned into the pCDNA3 vector, and the respective shRNA constructs were cloned in the pSUPER vector (three shRNAs per gene). The cells were seeded into 6-well plates at a density of 1 × 10^6^ cells/well, and were cultured overnight, followed by transfection with 2 µg plasmid or shRNA and the respective empty vectors using Lipofectamine™ 3000 (Invitrogen, California, USA) according to the manufacturer's guidelines.

### m^6^A dot blot analysis

Total RNA was extracted and purified using the miRNeasy Mini Kit (Qiagen #217004) and the GenElute™ mRNA Miniprep Kit (Sigma #MRN70), respectively. For control experiments (NB4, Kas-1), the rRNA was cleaned using the RiboMinus Transcriptome Isolation Kit (Invitrogen #K155002). In brief, mRNA was denatured and subjected to dot blot analysis using anti-m^6^A antibodies (1:2000, Synaptic Systems), as described previously 30. As a loading control, RNA spotted membrane was stained with 0.02% methylene blue (Sigma #1808) in 0.5 M sodium acetate (pH 5.0).

### Western blot analysis

Cells were washed twice with ice-cold PBS and lysed using ice-cold RIPA lysis buffer containing proteases. Western blot analysis of the cell protein lysates was performed as described previously 34. The following antibodies were purchased respectively from Cell Signaling Technology: anti-FTO (#31687), anti-RARA (#2554), anti-MYC (#9402), anti-METTL3 (#86132), anti-YTHDF2 (#80014), anti-FLT3 (#3462), anti-Bcl-2 (#15071), anti-Stat3 (#9139); from Abcam: anti-ALKBH5 (ab244296); from Santa Cruz: Anti-β-Actin (#sc47778); and from Thermo Fisher: Anti-puromycin (#BP2956).

### mRNA degradation and protein translation assays

The transfected and drug-treated cells were treated with 5 µg/mL actinomycin-D (Cayman Chemical #11421) for the indicated time points. Total RNA was purified using TRIzol® RNA isolation reagent (Invitrogen #15596018). The value was recorded as the percentage of mRNA remaining compared with the amount before actinomycin-D (Cayman Chemical #11421) treatment. Data were normalized to the levels of β-actin. For protein translation assays, NB4 and Kas-1 cells were treated with 200 ng/mL puromycin (Thermo Fisher #BP2956) and further incubated for the indicated time points. Then, cells were lysed for Western blot analysis and the relative protein expression was normalized to β-actin (1:1000, Santa Cruz #sc47778).

### Gene-specific m^6^A qPCR

To assess the relative abundance of specific mRNA transcripts in m^6^A IP and input groups, qPCR was performed. m^6^A RNA immunoprecipitation (MeRIP) was performed with Methylated RNA Immunoprecipitation (MeRIP) Kit (Bes5203, BersinBio) according to the manufacturer's guidelines. Reverse transcription and qPCR were performed with a QIAGEN's RT kit and 2 × SYBR green qPCR Master Mix. Cycle threshold (Ct) values were used to determine the relative enrichment of mRNA.

### AML patient samples

Diagnoses of AML were made according to the criteria of World Health Organization. For *in vivo* treatment, primary cells from AML and CML patients with >80% blasts were cultured in RPMI-1640 containing 20% FBS. All patients signed informed consent, which was approved by the Institutional Review Board before entering the study.

### Animal experiments

C57BL/6N mice, NU/NU Nude mice and immunodeficient NOD SCID mice (all male, 4-6 weeks old) were purchased from the Beijing Charles River Company (Beijing, China). All animals were managed as per the Guide for the Care and Use of Laboratory Animals. Animal experiments were approved by the Animal Care and Use Committee of the Changchun University of Chinese Medicine. Animal models were established as described previously 29, 35. In brief, for C1498 and FLT3+ models 0.5×10^6^ C1498 cells or transfected FLT3+ cells were injected into the tail-vein of C57BL/6N mice for the development of leukemic disease. For the “human in mouse” model, primary AML cells (10×10^6^) were injected into the tail-vein of NOD SCID mice. Upon the development of leukemic disease (established using a white blood cell (WBC) count), 0.1 mg/kg or 0.5 mg/kg of SsD was intraperitoneally injected three times per week for three consecutive weeks. For the WBC count, 2 μL of mouse tail-vein blood was mixed with 38 μL of Turk blood dilution fluid (Ricca Chemical, Arlington) and WBCs were counted under a microscope. For survival studies, mice were sacrificed when they showed any sign of distress, such as breathing disorders, weight loss, or immobility. Spleen weight and number of metastatic nodules were determined at the end of each experiment. For the Kas-1 nilotinibR animal model, parental or resistant cells were subcutaneously injected into the right flanks of nude mice. Then, tumor-bearing mice received three intraperitoneal injections of either 0.5 mg/kg nilotinib or 1 mg/kg SsD per week. Mice were sacrificed on the indicated day after resistant cell inoculation and tumors were harvested for H&E and IHC staining as well as a dot blot assay.

### Hematoxylin and Eosin (H&E), Immunohistochemical (IHC) and Giemsa staining

Histopathological staining was performed as previously described 36. Briefly, tissues collected during animal studies were fixed with 10% neutral buffered formalin and embedded in paraffin. The paraffin-embedded tissue specimens were sectioned into the size of 5 μM and stained with H&E. For IHC staining, the anti-PCNA primary antibody (Cell Signaling Technology, #12727) was used. Images were analyzed using Image-Pro Plus software. Giemsa staining assay was performed as previously described 29.

### Statistical analysis

All quantitative data are presented as the mean ± standard deviation (SD) of at least three independent experiments. The statistical analysis was performed using the Student's *t*-test. Survival curves were estimated using the Kaplan-Meier method, and significant differences between the survival curves were assessed by the Log-rank test. All analyses were performed using GraphPad Prism 7 software. A two-sided *p*-value of < 0.05 was considered statistically significant.

## Results

### SsD exhibits anti-proliferation activity in AML

**Figure 1:**
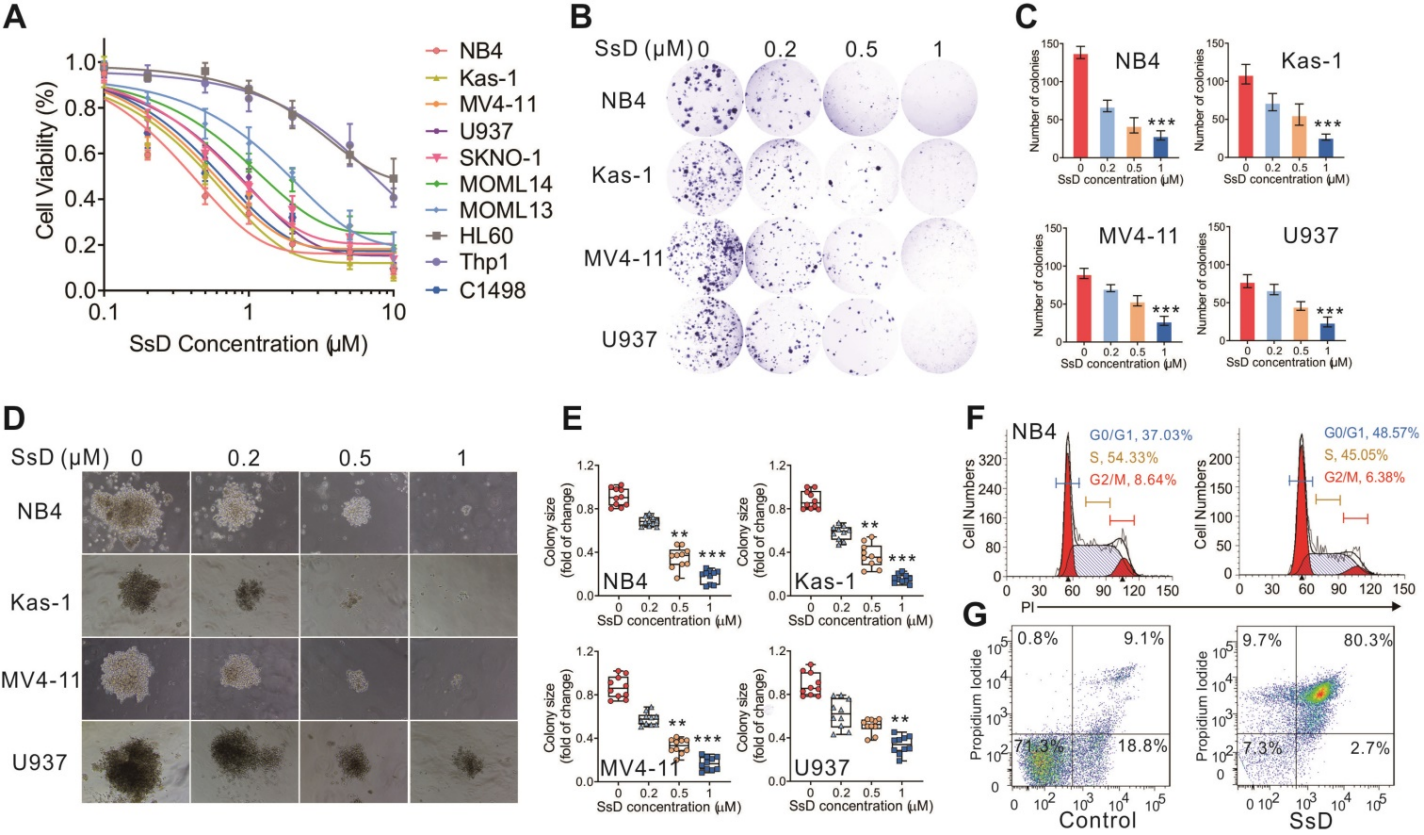
** SsD exhibits anti-proliferation activity and promotes myeloid differentiation and apoptosis.** (A) CCK-8 assays in SsD-treated AML cells after 48 h of treatment. (B) Colony-forming assays in NB4, Kas-1, MV4-11, and U937 cells, treated with different concentrations of SsD. Images show representative plates and (D) clones. (C) Graphs showing the colonies numbers from 3 independent experiments and (E) quantification of clone sizes from 10 clones. Data are the mean ± SD; *P < 0.05, **P < 0.01, ***P < 0.001. (F-G) Determination of the effect of SsD on cell-cycle arrest and apoptosis using FACS based on PI staining in NB4 cells after 48 h of treatment.

To elucidate the pharmacological effect of SsD in leukemia, we explored the effects of a series of SsD concentrations in 10 human leukemia cell lines. We found that SsD inhibits cell viability in a dose-dependent manner in most of these leukemia cell lines (Figures 1A). To further examine this inhibitory effect of SsD in leukemia, the colony assay in four leukemia cell lines, NB4, Kas-1, MV4-11, and U937 (top four sensitive cells to SsD) was carried out. Similar to the cell viability results, SsD significantly suppressed both colony number and size in the cell lines tested (Figures 1B-E). Interestingly, the SsD-induced inhibition of cell proliferation and viability was likely due to cell-cycle arrest in the G1 stage and increased apoptosis in NB4 cells (Figure 1F-G, and Figure S1A).

### Identification of SsD response-associated genes and pathways

**Figure 2:**
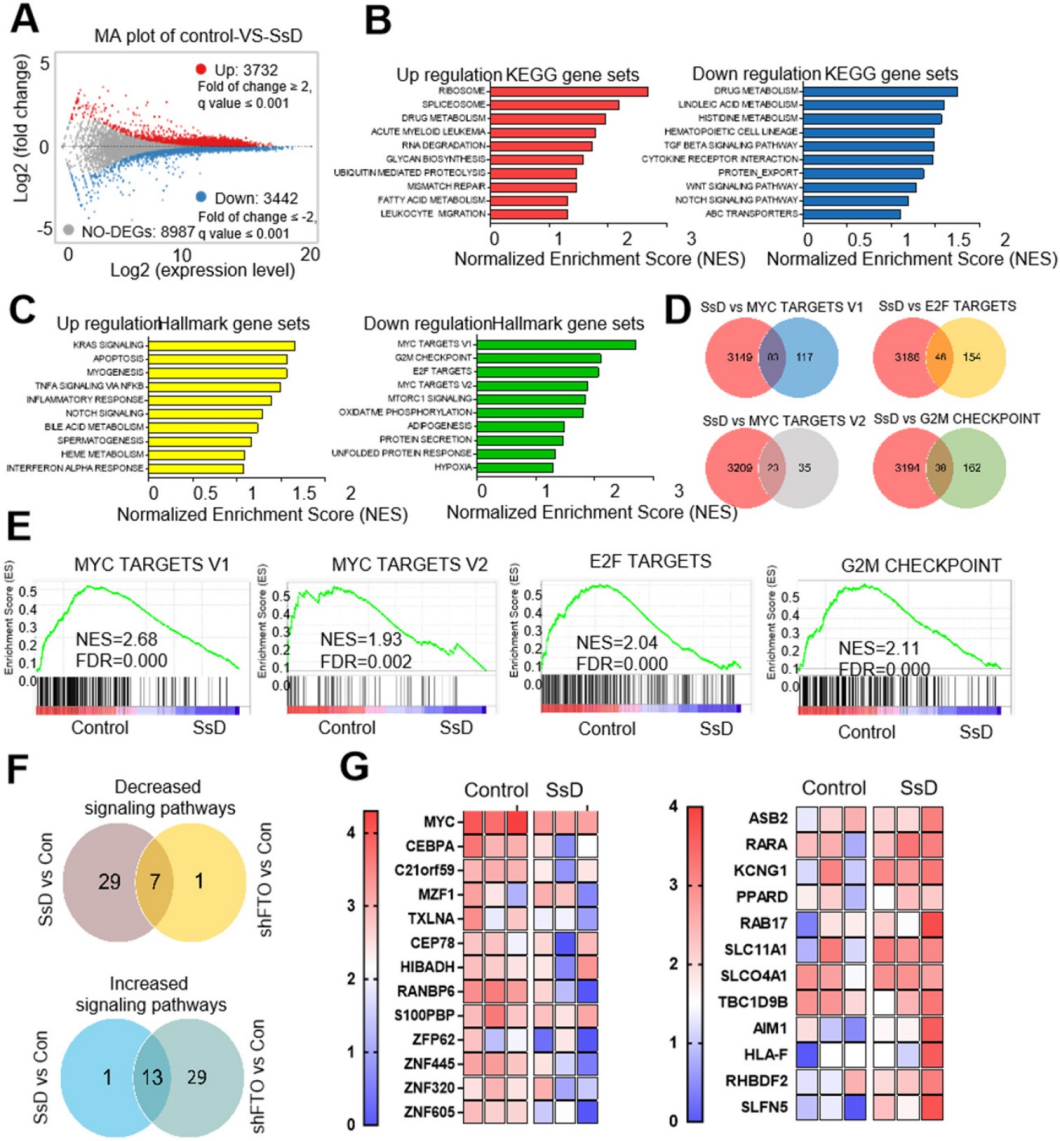
** Identification of genes and pathways related to the SsD response.** (A) Representative scatter plot of 7174 genes in control vs SsD-treated NB4 cells, 3732 upregulated genes are marked in red while 3442 downregulated genes are marked in blue. (B) KEGG pathway analysis of both downregulated and upregulated genes (based on the RNA-seq results) in control vs SsD-treated NB4 cells. P-value was corrected by FDR, and the FDR < 0.01 was considered significantly enriched. (C) Similarly, Gene Set Enrichment Analysis (GSEA) of pathways for the downregulated and upregulated genes in control vs SsD-treated NB4 cells. The P-value was corrected by FDR, and the FDR < 0.01 was considered significantly enriched. (D) Venn diagram showing the core genes of SsD-suppressed signaling pathways and the four shared signaling pathways in MSigDB database. (E) The top four signaling pathways suppressed by SsD in NB4 cells. (F) Venn diagram showing up- or downregulated genes in signaling pathways after SsD treatment or knockdown-mediated FTO inhibition. (G) Heat map analysis for significantly down-regulated or up-regulated gene in SsD treated NB4 cells.

To further determine the potential target that could be related to SsD sensitivity in leukemic cells, we performed RNA sequencing (RNA-seq) in SsD-treated NB4 cells which is the most sensitive leukemia cell line. Differentially-expressed genes (DEGs) were identified. Genes with a fold change of > 2 were classified as DEGs. Based on this criterion, we found a total of 3732 upregulated and 3442 downregulated genes (Figure 2A and Table S1). To reveal the potential underlying mechanisms of SsD, we next performed enrichment analyses and investigated the DEG-related pathways. We screened for the top ten pathways that were enriched with up-regulated and down-regulated genes (Figure 2B and Table S2). In addition, GSEA was used to investigate the signaling pathways affected by SsD treatment (Figure 2C and Table S3). Our data revealed that SsD treatment caused significant suppression of MYC targets, E2F targets, and G2M checkpoint signal cascades. Interestingly, these findings agreed with the suppressed signaling pathway by FTO inhibitors and the knockdown of endogenous FTO (FTO KD). Therefore SsD inhibitory effects may contribute to FTO inhibition on the cell cycle and proliferation (Figure 2D-E and Table S4). Furthermore, the vast majority of pathways upregulated by FTO knockdown were enriched for SsD, and similarly, the majority of SsD-suppressed signaling pathways were also inhibited by FTO knockdown (Figure 2F and Table S5). More importantly, SsD mediated changes in m^6^A-related genes (downregulated or upregulated) were significantly consistent (Figure 2G and Table S6). Overall, these findings indicated that the SsD might be involved in the FTO-mediated m^6^A RNA methylation pathway.

### FTO is a direct target of SsD

**Figure 3:**
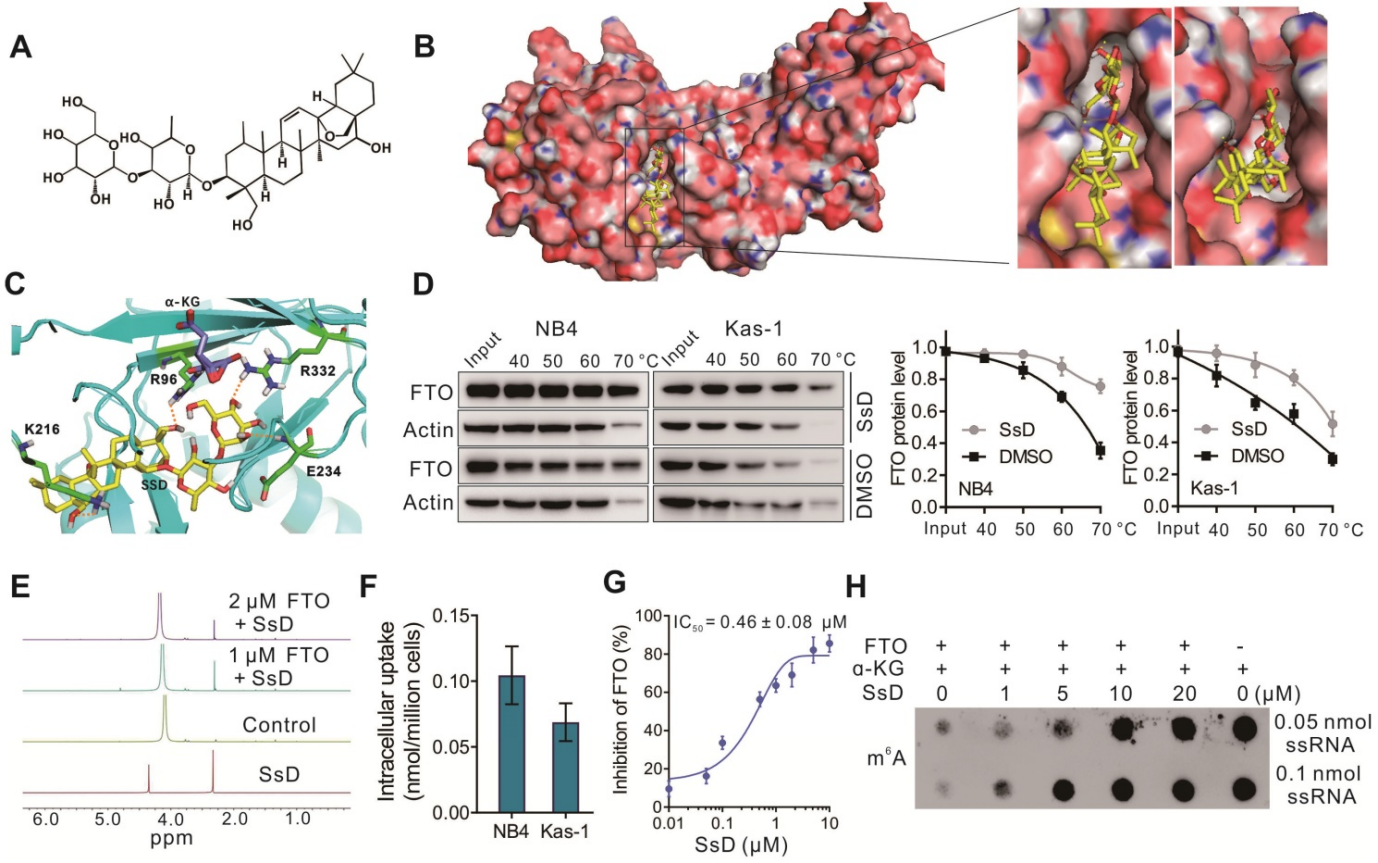
** FTO is a direct target of SsD.** (A) The molecular structure of SsD is shown. (B) Molecular docking illustrations showing the potential binding pocket of SsD in FTO. (C) Structural complex of FTO bound with SsD. The surrounding amino acids and α-KG are shown. (D) Western blots showing the effects of 1 µM SsD on the thermal stabilization of FTO protein. CETSA was assayed in cell lysates. The results were derived from three biological replicates. (E) NMR measurement of SsD interaction with FTO. (F) Determination of cellular uptake of SsD by LC-MS/MS quantitation. AML cells were treated with 1 µM SsD for 48 h. (G) *In vitro* quantification of inhibition by SsD on FTO demethylation activity of m^6^A in RNA using HPLC. (H) In a cell-free system, dot blot analysis of m^6^A abundance in the presence of various SsD concentrations and FTO protein is shown.

Based on gene microarray data that SsD-mediated inhibition of leukemogenesis was mainly due to the m^6^A pathway, we hypothesized that SsD might modulate m^6^A RNA methylation in leukemia. Previously it was reported that FTO plays the ontogenetic role in leukemogenesis 37, therefore, we examined FTO expression in all ten cell lines used in this study (Figure S1B). We found that FTO expression in these cell lines was consistent with the sensitivity of the cell growth inhibition by SsD. To validate the direct binding of SsD to FTO, we firstly performed molecular docking analysis based on the published crystal structure of FTO (PDB ID: 6AKW). The optimum binding site for SsD showed an extraordinary shape that was complementary with the substrate-binding site, occupying the entire binding pocket (Figure 3A-B). Four amino acid residues (R96, K216, E234, R332) were involved in the interaction with SsD (Figure 3C), which plays key roles in the inhibition of FTO activity as previously reported 38. Next, we investigated the interaction between FTO and SsD by a variety of biochemical experiments. Cellular thermal shift assays (CETSAs) data suggested that SsD binds to FTO rather than ALKBH5 (Figure S1C-D). In NB4 and Kas-1 AML cells, SsD binding to FTO protein induced an obvious thermal shift, suggesting protection from the thermal degradation (Figure 3D). We also verified the activity of SsD against ALKBH5 in a cell-free system by dot blot analysis. As shown in Figure S1E, we did not find a considerable SsD-mediated change of ALKBH5 activity. The interaction between FTO and SsD was further investigated using NMR where a dose-dependent attenuation of signals was observed in titrations (Figure 3E), and negative saturation transfer difference signals were also detected (Figure S1F). These results indicated that FTO interfered with the state of SsD. Next, we analyzed the cellular uptake of SsD using LC-MS/MS quantitation (Figure 3F), notably, SsD was detected around 0.05-0.14 nmol/million cells in NB4 and Kas-1 cells. Inhibitory activity of SsD on FTO demethylation *in vitro* is shown using HPLC (Figure 3G). Furthermore, in a cell-free system, dot blot assays verified the SsD-mediated competitive suppression of FTO activity (Figure 3H). Collectively, the aforementioned results strongly indicated that FTO is a direct target of SsD and could be the main mediator of SsD-induced growth-suppressive effects in leukemic cells.

### SsD induces m^6^A modification by directly inhibiting FTO m^6^A demethylation activity

**Figure 4:**
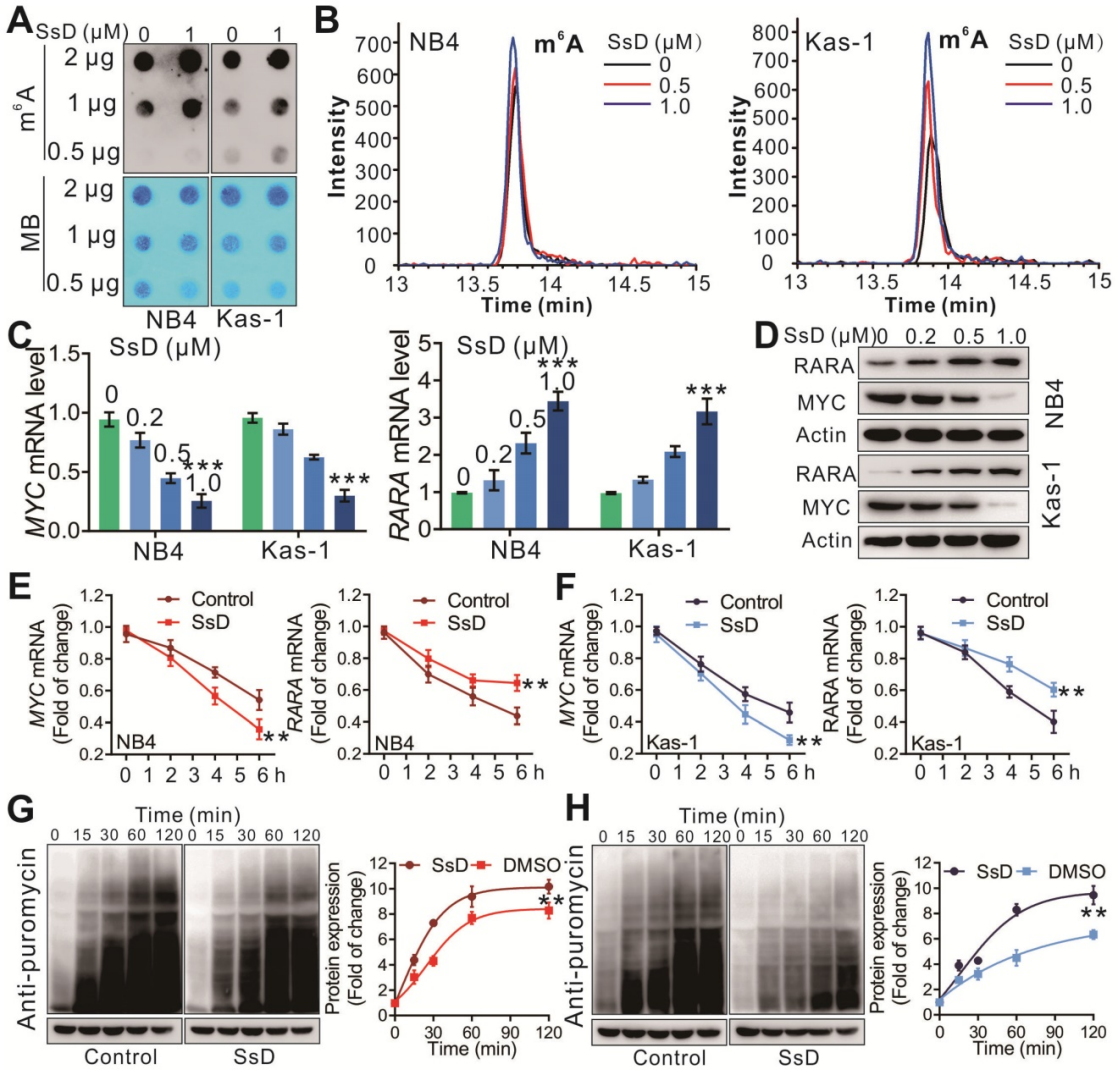
** SsD induces m^6^A modification.** (A) m^6^A dot blots of indicated cells treated with SsD are shown. MB (methylene blue) represents the loading control of RNA samples. (B) TSQ traces of FTO demethylation of m^6^A in ssRNA in the absence and presence of the SsD in NB4 and Kas-1 cells are shown, respectively. (C) Expression levels of indicated genes in cells treated with SsD measured using qPCR. Data represent three independent experiments. (D) Western blot analysis of the protein levels of indicated genes in SsD treated cells. (E-F) qPCR analysis of NB4 and Kas-1 cells treated with SsD for 24 h, followed by 5 µg/mL actinomycin-D treatment for the indicated time points. Gene expression was normalized to GAPDH. (G-H) Western blot analysis for protein stability in cells treated with SsD for 24 h, followed by 200 nM puromycin treatment for the indicated time points. Graphs are showing the quantification of Western blot analysis normalized to β-actin; Data represent three independent experiments and present the mean ± SD; *p < 0.05, **p < 0.01, ***p < 0.001.

To test the changes in m^6^A on RNA, we performed the m^6^A dot blot assay in SsD treated leukemia cells. As shown in Figure 4A and S2A, SsD substantially increased the abundance of m^6^A in transcriptomes. Furthermore, HPLC-MS/MS quantitation confirmed the increase of cellular m^6^A in mRNA of SsD-treated NB4 and Kas-1 cells (Figure 4B and S2B). Next, we checked the expression of *MYC* and *RARA*, two direct targets of FTO in SsD-treated cells. SsD notably downregulated MYC but upregulated RARA in NB4 and Kas-1 cells both at the mRNA and protein level (Figure 4C-D). Interestingly, an SsD-dependent decrease in MYC and RARA was due to decreased mRNA and protein stability in FTO-overexpressed cells (Figure 4E-4H). Gene-specific m^6^A qPCR to detect the m^6^A methylation status of MYC and RARA was performed and the results indicated that a decrease of MYC and RARA mRNAs stability was a consequence of an alteration of m^6^A methylation in these genes (Figure S2C). We also observed that FTO overexpression rescued RARA upregulation and MYC suppression by SsD treatment, thereby indicating that MYC and RARA are the FTO-dependent downstream targets of SsD (Figure S2D). There was no change in the stability of ALKBH5, METTL3, YTHDF2 proteins (Figure S2E). Altogether, these results further demonstrated that FTO and its downstream targets (e.g., MYC, RARA) are the major effectors of SsD in FTO overexpressed leukemia cells. We also showed that small hairpin RNAs (shRNAs) based on FTO knockdown reinstated the effects of SsD on increasing global m^6^A levels of cellular poly(A)+ RNA in NB4 cells (Figure S2F). Of note, forced expression of wild-type FTO, but not mutant FTO significantly promoted the sensitivity of Thp1 cells to R-2HG, which indicated that R96, K216, E234, and R332 mutations could disrupt the enzymatic activity of FTO (Figure S2G and S2H). The panel experiments of pharmacological or genetic inhibition by FTO inhibitor R-2HG or shRNA confirmed that SsD treatment had similar phenotypes (Figure S2I-N). These results further strengthened the idea that SsD possessed FTO-mediated m^6^A RNA methylation activity.

### SsD significantly inhibits progression of AMLs *in vivo*

**Figure 5:**
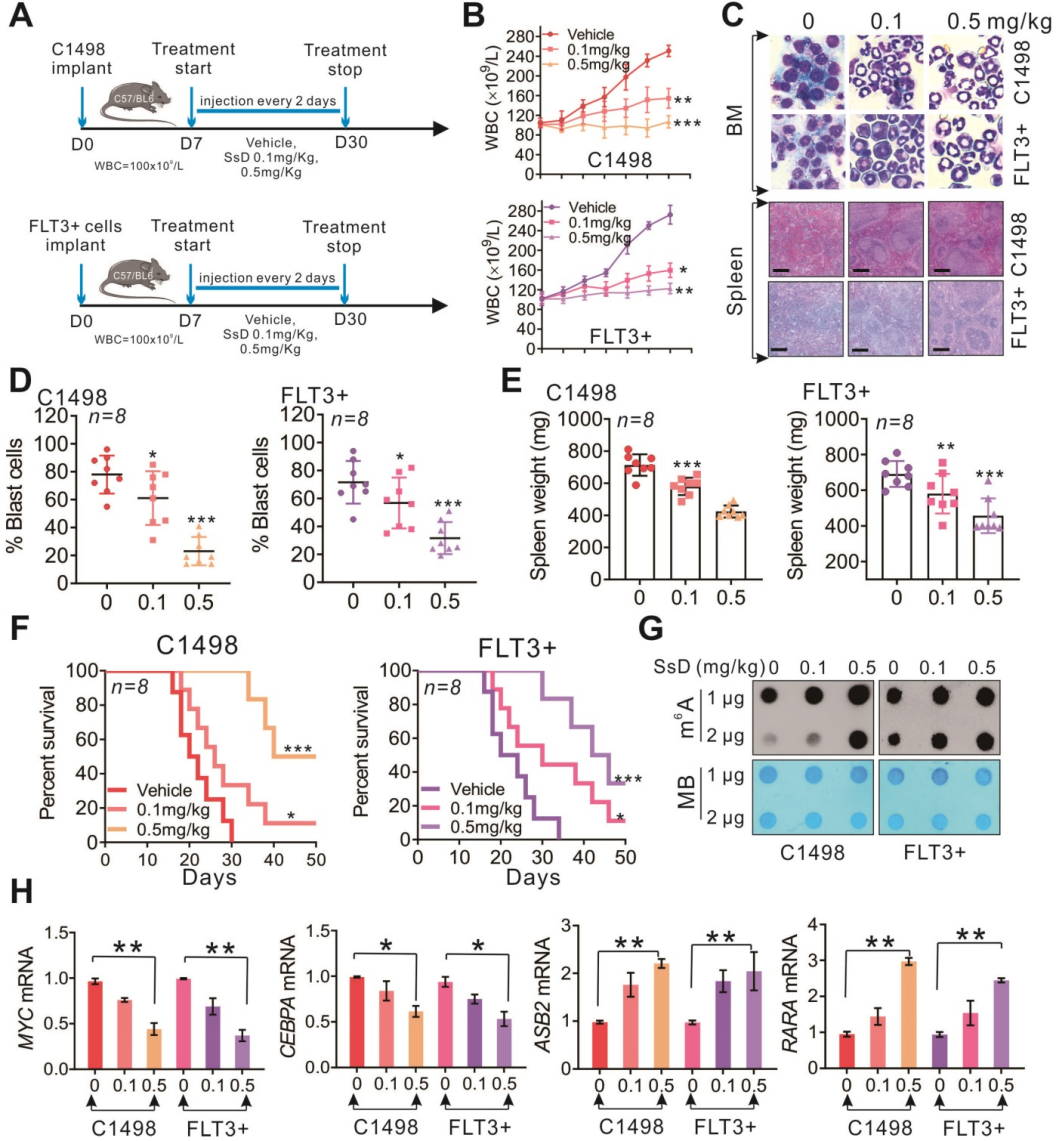
** SsD significantly inhibits the progression of sensitive AMLs *in vivo*.** (A) Leukemic mice were developed by intravenous injection of C1498 and FLT3+ cells into 4 weeks old C57BL/6 mice. The WBC count was used as an indicator for the development of leukemic disease. SsD or vehicle was injected 3 times a week for 3 weeks. (B) WBC count of leukemia-bearing mice (n = 8) is shown. (C) Images show representative external views of the spleen with histological analysis of H&E-stained spleen sections (Scale bars, 1 cm, lower) and Wright-Giemsa-stained bone marrow (BM) cells (upper). (D-E) The quantification for blast cells and spleen weight is shown. (F) The survival curve of leukemia-bearing mice was calculated by Kaplan-Meier survival analysis (n = 8). (G) Using the dot blot assay, m^6^A abundance was determined in the RNA samples in SsD-treated primary BM cells at 48 h. The results were obtained from two biological replicates. (H) qPCR expression analysis of *MYC, CEBPA, ASB2,* and* RARA* in BM. Data are the mean ± SD; *P < 0.05, **P < 0.01.

To investigate the therapeutic efficiency of SsD against leukemia *in vivo*, C1498, and FLT3+ leukemia mouse models were established (Figure 5A and S3A-B). The vehicle group showed rapid WBC growth, and total WBC in this group expanded to 250 ± 10×10^9^/L in C1498 mice or 272 ± 13×10^9^/L in FLT3+ mice within 30 days. Administration of 0.1 mg/kg or 0.5 mg/kg doses of SsD significantly inhibited WBC growth to an average WBC of 154 ± 17×10^9^/L and 160 ± 11×10^9^/L, or 10^7^ ± 11×10^9^/L and 122 ± 7×10^9^/L respectively (Figure 5B). Consistent with the inhibitory effects on WBC, SsD also efficiently impaired the bone marrow (BM) clonogenic potential (blast %) (Figure 5C and 5D). Moreover, spleens isolated from the SsD-treated group weighed less compared to those from control groups, indicating that SsD treatment markedly reversed the splenomegaly and suppressed the metastatic tumor cells in the spleen (C1498 mice: vehicle, 713 ± 62 mg; 0.1 mg/kg, 580 ± 51 mg; 0.5 mg/kg, 424 ± 34 mg; FLT3+ mice: vehicle, 691 ± 68 mg; 0.1 mg/kg, 581 ± 104 mg; 0.5 mg/kg, 457 ± 91 mg) (Figure 5C and 5E). Furthermore, lung metastatic nodules in the SsD group were significantly lower (in numbers) than that of other groups (C1498 mice: vehicle, 15 ± 3.0; 0.1 mg/kg, 8.7 ± 2.2; 0.5 mg/kg, 4.3 ± 2.3; FLT3+ mice: vehicle, 14.5 ± 1.9; 0.1 mg/kg, 8.8 ± 2.8; 0.5 mg/kg, 4.8 ± 1.6), further indicating the less aggressive leukemic growth in lung (Figure S3C and S3D). Evidently, in agreement with the pathological phenotype, SsD-treated mice also had a significantly longer survival time (Figure 5F). However, we did not observe obvious alterations in mouse body weight (Figure S3E), food intake, or mobility, suggesting minimal side effects of SsD in the tested settings. Mechanistically, the anti-leukemic effects of SsD *in vivo* were mediated by its m^6^A RNA methylation activities (Figure 5G); since BM from SsD-treated mice had increased m^6^A RNA methylation leading to modulate target genes (Figure 5H). Next, to test the *in vivo* toxicity of SsD, we administrated normal mice with PBS or SsD. No death or unusual behaviors were observed in SsD-treated mice during the entire experiment. Also, all major organs of mice in the SsD group, including heart, liver, spleen, lung, and kidney showed no pathological changes even after one month-administration of SsD at a high dose of 0.5 mg/kg (Figure S4).

### SsD inhibits patient primary cells

**Figure 6:**
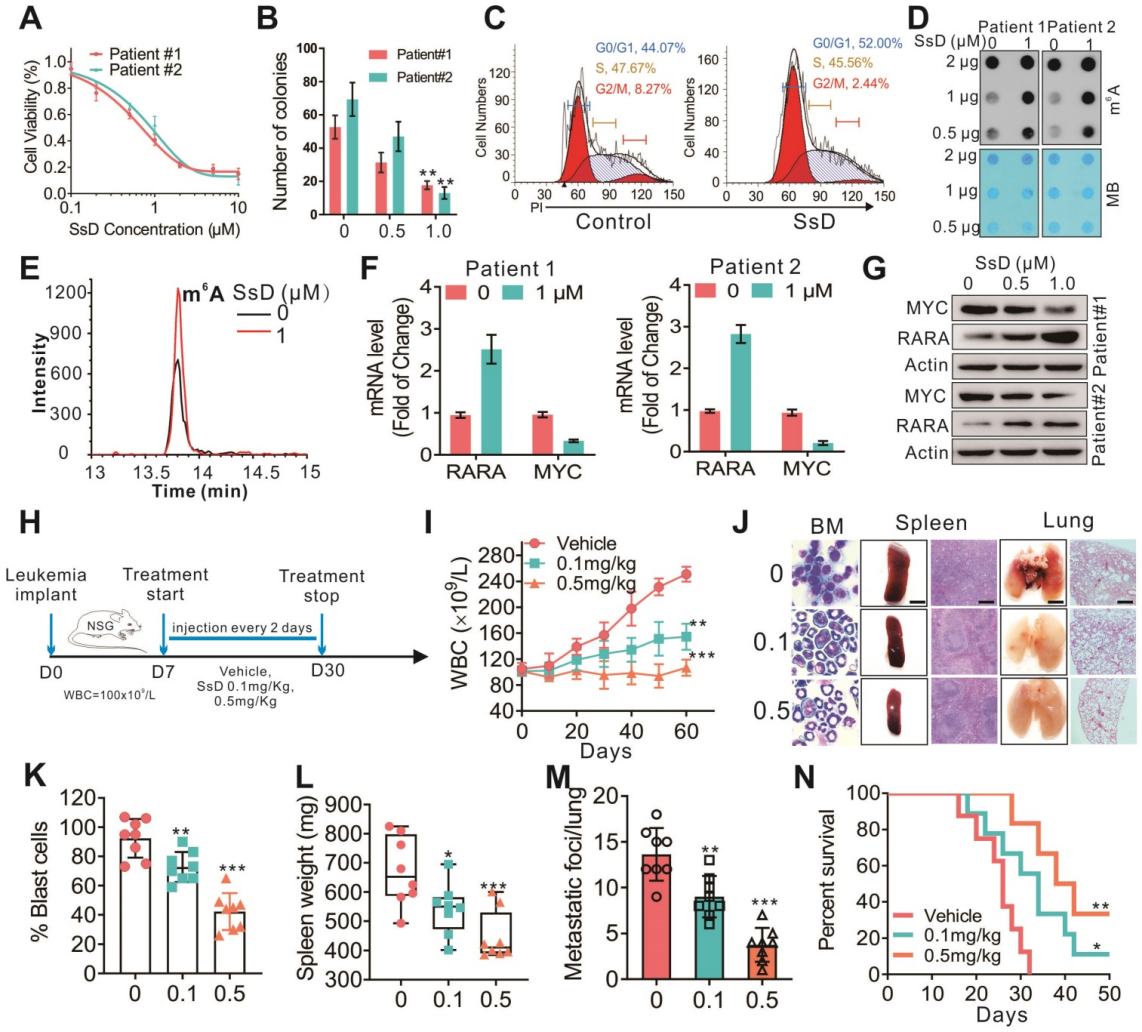
** SsD inhibits patient primary cells.** (A) CCK-8 assays in the cells derived from two patients treated with SsD for 48 h. (B) Colony-forming assays of patient cells treated with SsD. Graphs showing the colony number from 3 independent experiments. Data are the mean ± SD; *P < 0.05, **P < 0.01. (C) Effect of SsD on cell-cycle arrest was determined using FACS based on PI staining in patient cells after 48 h of treatment. (D) After 48 h of SsD treatment, m^6^A abundance in the RNA samples was determined in primary patient cells using a dot blot assay. (E) TSQ traces of FTO demethylation of m^6^A in ssRNA in the absence and presence (1 µM SsD for 48 h) of SsD in patient cells. (F) qPCR expression analysis of the indicated genes in patients' SsD-treated cells. Data represent three independent experiments. (G) Western blot analysis of the indicated genes in patients' SsD-treated cells. (H) Leukemia-bearing mice were developed by intravenous injection of patient cells into 4-week old NSG mice. (I) WBC count of leukemia-bearing mice (n = 3) is shown. (J) Images show representative external views of the spleen with the histological analysis of H&E-stained spleen sections (Scale bars, 1 cm, middle), Wright-Giemsa-stained bone marrow (BM) cells (left), and external views of the lung with H&E-stained sections (right) from leukemia-bearing mice. (K-M) The quantification of blast cells (K), spleen weight (L), and tumor growing nodules on lung (M) are shown. (N) The survival curve of leukemia-bearing mice calculated by Kaplan-Meier survival analysis (n = 5) is shown.

Next, peripheral blood from AML patients (n=3) was obtained from the Tumor Tissue/Biospecimen Bank of the First Hospital of Jilin University (Changchun City, Jilin Province, China) to further evaluate the effect of SsD on leukemia progression. The primary cells were handled and cultured as previously described 39. In agreement with the results obtained from cell lines experiments, here too, SsD significantly suppressed the leukemia cell proliferation (Figure 6A), colony-formation ability (Figure 6B), induced cell-cycle arrest (Figure 6C) and increased cell apoptosis (Figure S5A-C). Mechanistically, this was regulated by FTO-mediated m^6^A RNA methylation and its targets in SsD-treated primary cells (Figure 6D-G and S5D). More importantly, when we used “human-in-mouse” xeno-transplantation leukemic models to evaluate the effect of SsD on leukemia progression *in vivo* (Figure 6H), similarly as above, treatment with SsD significantly inhibited AML progression, including a lower WBC, decreased leukemic blasts in BM, reduced splenomegaly, inhibited lung metastasis, and prolonged survival in mice xenotransplanted with AML primary cells (Figure 6I-N). Moreover, we did not observe any obvious alterations in mouse body weight (Figure S5E). Thus, these *in vivo* findings suggested that SsD holds therapeutic potential to treat FTO-mediated AML.

### SsD overcomes N^6^-methyladenosine-mediated leukemia resistance to tyrosine kinase inhibitors

**Figure 7:**
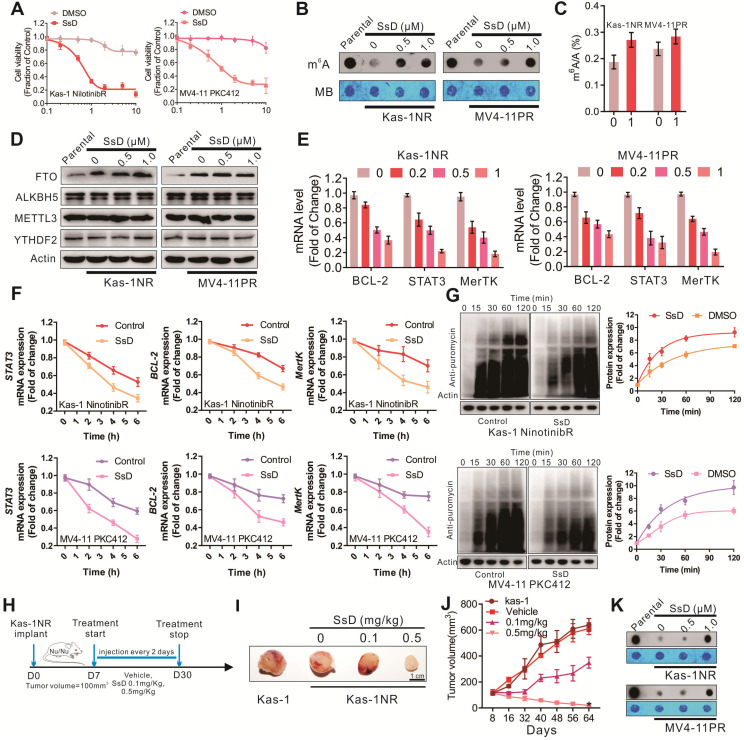
** SsD overcomes N6-methyladenosine-mediated leukemia resistance to tyrosine kinase inhibitors.** (A) CCK-8 assays in resistant cells treated with SsD for 24 h. (B) Dot blot analysis of m^6^A abundance in the RNA samples in parental resistant cells after 48 h of SsD treatment is shown. (C) Quantitation of m^6^A/A in mRNA in resistant cells treated with 1 µM SsD for 48 h was performed using LC-MS/MS. (D) Western blot analysis of indicated genes in resistant cells treated with SsD is shown. (E) qPCR expression analysis of indicated genes in resistant cells treated with SsD is shown. Data represent three independent experiments. (F) qPCR analysis of Kas-1NR and MV4-11PR cells treated with SsD for 24 h, followed by 5 µg/mL actinomycin-D treatment for the indicated time points is shown. The gene expression levels were normalized to GAPDH. (G) Western blot analysis of anti-puromycin in cells treated with SsD for 48 hours, followed by 200 nM puromycin treatment for the indicated time points is shown. Graphs are the quantitative representation of Western blot results normalized to β-actin. (H) Leukemia-bearing mice were developed by intravenous administration of Kas-1 or Kas-1NR (0.5×10^6^) cells into 4-week old mice (three times a week for three weeks). (I) Images of tumors and (J) quantification of tumor growth are shown. (K) m^6^A dot blot analysis in tumors is presented.

Incapacitating the requirement of FTO-dependent m^6^A demethylation could yield a therapeutic opportunity to prevent the development of TKI resistant phenotypes. Since FTO inhibition sensitizes resistant cells to TKI treatment, we examined the efficacy of SsD in TKI-resistant cells. For *in vitro* examination, Kas-1 nilotinib resistant cells (Kas-1NR) and MV4-11 PKC412 (MV4-11PR) were established as reported previously 30. Initially, these cells were exposed to 1 μM SsD for 6 h to suppress FTO activity, followed by 72 h treatment with nilotinib or PKC412. We found that SsD in combination with nilotinib or PKC412 was much more effective in lowering cell viability (Figure 7A), indicating that SsD induced pronounced inhibition of cell proliferation in Kas-1NR and MV4-11PR cells. Molecular characterization of cells also revealed that TKIs-mediated m^6^A hypomethylation was rescued (Figure 7B-C, and S6A). Though SsD did not change the protein levels of ALKBH5, METTL3, YTHDF2 in Kas-1NR, and MV4-11PR cells (Figure 7D), TKIs-upregulated expression of m^6^A-associated genes, *MerTK*,* BCL-2,* and *STAT3,* was impaired (Figure 7E and S6B). Consistent with the above-mentioned results, *MerTK*, *BCL-2*, and *STAT3* were found to more stable both at mRNA and protein levels in TKI-resistant cells. However, SsD treatment decreased the stability of *MerTK* and *BCL-2* transcripts, which subsequently led to their decreased translation (Figure 7F and 7G). Gene-specific m^6^A qPCR confirmed that changes in RNA or protein levels of *MerTK*, *BCL-2*, and *STAT3* were caused by m^6^A-mediated mRNA stability. To examine these effects *in vivo*, we engrafted nude mice with Kas-1 nilotinib resistant cells. After cell inoculation, to retain resistance phenotypes, we continued administrating nilotinib intraperitoneally twice a week until the tumor volume approached 100 mm^3^. Then, randomized groups were treated with suboptimal doses of SsD (0.1 mg/kg, 0.5 mg/kg) (Figure 7H). SsD itself marginally slowed down resistant tumor growth, as demonstrated by the smallest tumor volume (Kas-1, 639 ± 49 mm^3^; Vehicle, 611 ± 45 mm^3^; 0.1 mg/kg, 348 ± 42 mm^3^; 0.5 mg/kg, 20 ± 8 mm^3^) (Figure 7I and 7J). Mechanistically, we observed an overall increase in mRNA m^6^A methylation (Figure 7K). Furthermore, PCNA-stained sections were evaluated to confirm the anti-cancer efficiency of SsD (Figure S6D). As previously, we did not observe any alterations in mouse body weight (Figure S6E).

### Saikosaponin A (SsA) exerts similar effects to SsD

SsA, the enantiomer of SsD is also one of the major active components of Bupleurum falcatum, which is structurally and chemically related to SsD (Figure S7A). Similarly, it possesses numerous pharmacological activities, including immunoregulatory, anti-inflammatory, and hepatoprotective activities 40. We studied whether it had effects that were identical on AML as SsD *in vivo* and *in vitro*. As shown, SsA also inhibited colony number and induced cell-cycle arrest at the G1 stage and apoptosis (Figures S7B-D), but compared with SsD, it was less powerful. In terms of the mechanism of action, SsA increased global m^6^A modification by directly inhibiting the demethylation activity of FTO (Figures S7E-H). We found that SsA also displayed a growth-suppressive effect in AML cells both *in vitro* and *in vivo* (Figures S7I-O). However, significantly inhibitory effects on AML proliferation were observed with SsD compared to SsA.

## Discussion

AML is one of the most common leukemias, characterized by differentiation arrest and uncontrolled proliferation of malignant blasts. The treatment of AML is still limited due to drug resistance and side effects of existing drug, and novel therapeutic strategies are urgently needed 28. Numerous natural products originated from Chinese herbal medicine exhibit anti-cancer activities. Epigallocatechin gallate (EGCG), Berberine, and Triptolide are reported to have a therapeutic effect in leukemia 41. SsD, an effective component of Radix Bupleuri, exhibits a promising anti-tumor effect in various types of cancer, including lung cancer, liver cancer, breast cancer, prostate cancer, etc. Recently, we reported that SsD-loaded by a nanocomposite effectively inhibited tumor growth and metastasis of breast cancer *in vitro* and *in vivo* through *VEGFR*, *AKT*, and *ERK* related to the angiogenic pathway 34. However, therapeutic effect and molecular mechanism of SsD in AML have rarely been reported. Here, we explored SsD as a potential treatment in leukemia. In agreement with our hypothesis, we found that SsD significantly inhibited AML leukemogenesis in a relative lower dose-dependent manner (0.5 to 1 µM).

We performed RNA sequencing in SsD-treated NB4 cells to determine the potential target that could be involved. Fascinatingly, we found that SsD treatment caused the significant suppression of MYC targets, E2F targets, and G2M checkpoint signal cascades, consistent with the downregulated signaling pathway by FTO inhibitors and FTO knockdown. More importantly, SsD significantly regulated downstream targets of FTO/m^6^A, such as *MYC*, *CEBPA*, *ASB2*, *RARA*, etc. These findings indicated that SsD could be involved in the FTO-mediated m^6^A RNA methylation pathway. Therefore, we speculated whether SsD could be used as a small molecule inhibitor targeting FTO to regulate the RNA methylation of m^6^A. FTO inhibitors have been reported to significantly promote the methylation of m^6^A, thereby inhibiting the proliferation of leukemia cells. The SsD/FTO binding experiments, such as AutoDock assay, CETSA, NMR measurement, FTO activity assay, to validate our hypothesis of using SsD as a target inhibitor of FTO.

Leukemia is frequently associated with activating mutations of receptor tyrosine kinases (RTKs), including BCR/ABL, KIT, FLT3, etc. To examine the effect of SsD *in vivo*, we performed studies in leukemia animal models as presented in our previous reports 35. C1498 is a typical AML model. FMS-like tyrosine kinase 3 (FLT3) is a receptor tyrosine kinase that is exclusively expressed in the hematopoietic compartment. One of the most common mutations in AML involves the internal tandem duplication (ITD) of FLT3, which occurs in ~25% of all newly diagnosed cases of AML with a poor prognosis 42. In this study, we also showed that SsD has a good effect on FLT3-ITD mutations bearing AML cells. This indicated that FLT3-ITD mutations might be positively correlated with FTO expression, which is consistent with the data presented in a previous report 18. In preclinical testing, these results suggest utilizing SsD against the FLT3-ITD mutant genetic AML subtype. The mechanism-based rationale for the correlation between FLT3 and FTO must be investigated further.

On the other hand, drug resistance is a major problem that must be resolved. Many tyrosine kinase inhibitors (TKIs) rapidly acquired resistance to TKIs presenting a major hurdle for successful leukemia treatment. Our findings emphasized the TKI-independent mechanism for the development of drug resistance in leukemia 43. Previously, we showed that the developing resistant phenotypes during TKI therapy depend on m^6^A reduction resulting from FTO overexpression in leukemia cells. Mechanistic investigations revealed that the FTO dependent m^6^A demethylation enhances mRNA stability of proliferation/survival transcripts bearing m^6^A and subsequently leads to increased protein synthesis. FTO inhibitor rhein or MA induced more pronounced inhibition of cell proliferation in TKI-resistant cells through enhancing m^6^A methylation with barely detectable changes in FTO protein expression leading to impaired expression of *COL18A1, CTSB, CITED2, MERTK, MTHFR,* and* BCL-2* 30. In the present study, SsD sensitized MV4-11 or Kas-1 resistant cells to nilotinib and PKC412 via rescuing FTO-mediated m^6^A hypomethylation, which in turn decreased the stability of MTHFR and BCL-2 transcripts and proteins, thereby supporting SsD as FTO inhibitor that overcomes TKI-independent-based resistance.

In conclusion, our studies reveal a previously unrecognized link between FTO/m^6^A-modification signaling and elucidate the function of SsD in AML. It is of importance to further test the combination of SsD with other molecule inhibitors in the clinic against AML, such as other FTO inhibitors, DNA hypomethylating agents, histone acetyltransferase/methyltransferase/deacetylase inhibitors, tyrosine kinase inhibitors or other cytotoxic chemotherapy drugs. We anticipate that this study may provide insight into a promising strategy for target FTO/m^6^A-based epitranscriptome and highlights the clinical potential of Saikosaponin for leukemia therapy.

## Supplementary Material

Supplementary figures.

Supplementary table S1.

Supplementary table S2.

Supplementary table S3.

Supplementary table S4.

Supplementary table S5.

Supplementary table S6.

Supplementary table S7.

## Abbreviations

SsD: Saikosaponin-d
m^6^A: N^6^-methyladenosine
AML: Acute Myeloid Leukemia
FTO: fat mass and obesity-associated protein
TKI: Tyrosine Kinase Inhibitor
FLT3: FMS-Like Tyrosine Kinase 3
IDH2: Isocitrate Dehydrogenase 2
IDH1: Isocitrate Dehydrogenase 1
ERBB2: EGFR/EGF receptor 2
BCL-2: B Cell Lymphoma-2
FDA: Food and Drug Administration
ALKBH5: alkB homolog 5
R-2HG: R-2-hydroxyglutarate
COX: cyclooxygenase
SUMO: sentrin/small ubiquitin-like modifier
SENP5: specific protease 5
Gli1: glioma-associated oncogene homolog 1
SERCA: sarco endoplasmic reticulum calcium adenosine triphosphatase
CEBPA: CCAAT/enhancer binding protein alpha
PI: propidium iodide
DMSO: Dimethyl Sulfoxide
DTT: Dithiothreitol
PBS: Phosphate-Buffered Saline
BSA: Bovine Serum Albumin
HPLC: High-Performance Liquid Chromatography
IC50: half maximal inhibitory concentration
ssRNA: single-stranded RNA
EDTA: Ethylenediaminetetraacetic acid disodium salt
RARA: retinoic acid receptor α
SDS-PAGE: sodium dodecyl sulfate polyacrylamide gel electrophoresis
PVDF: polyvinylidene difluoride
RT: room temperature
WBC: white blood cell
H&E: Hematoxylin and Eosin
IHC: Immunohistochemical
DEGs: differentially expressed genes
CETSAs: Cellular thermal shift assays
MERTK: Mer proto-oncogene tyrosine kinase
STAT3: Signal Transducer and Activator of Transcription 3
FLT3-ITD: FMS-like Tyrosine Kinase 3 -internal tandem duplication
BRD4: Bromodomain-containing protein 4
SOCS2: suppressor of cytokine signaling 2
EGFR: epidermal growth factor receptor
SsA: Saikosaponin A
ShRNAs: small hairpin RNAs
GSEA: Gene Set Enrichment Analysis
YTHDF2: YTH-domain family 2
BM: bone marrow
PI: propidium iodide
DAPI: 4',6-diamidino-2-phenylindole
R-2HG: R-2-hydroxyglutarate

## References

[1] Short Nicholas J, Rytting Michael E, Cortes Jorge E (2018). Acute myeloid leukaemia. The Lancet.

[2] Wu G, Liu T, Li H, Li Y, Li D, Li W (2018). c-MYC and reactive oxygen species play roles in tetrandrine-induced leukemia differentiation. Cell death & disease.

[3] Yu Z, Du J, Hui H, Kan S, Huo T, Zhao K, Wu T, Guo Q, Lu N (2021). LT-171-861, a novel FLT3 inhibitor, shows excellent preclinical efficacy for the treatment of FLT3 mutant acute myeloid leukemia. Theranostics.

[4] Kozono S, Lin YM, Seo HS, Pinch B, Lian X, Qiu C (2018). Arsenic targets Pin1 and cooperates with retinoic acid to inhibit cancer-driving pathways and tumor-initiating cells. Nat Commun.

[5] Stone RM, Mandrekar SJ, Sanford BL, Laumann K, Geyer S (2017). Midostaurin plus Chemotherapy for Acute Myeloid Leukemia with a FLT3 Mutation. The New England journal of medicine.

[6] Stein EM, Fathi AT, DiNardo CD, Pollyea DA, Roboz GJ (2020). Enasidenib in patients with mutant IDH2 myelodysplastic syndromes: a phase 1 subgroup analysis of the multicentre, AG221-C-001 trial. The Lancet Haematology.

[7] Choe S, Wang H, DiNardo CD, Stein EM, de Botton S, Roboz GJ (2020). Molecular mechanisms mediating relapse following ivosidenib monotherapy in IDH1-mutant relapsed or refractory AML. Blood advances.

[8] Baccelli I, Gareau Y, Lehnertz B, Gingras S, Spinella JF, Corneau S (2019). Mubritinib Targets the Electron Transport Chain Complex I and Reveals the Landscape of OXPHOS Dependency in Acute Myeloid Leukemia. Cancer Cell.

[9] Sharon D, Cathelin S, Mirali S, Di Trani JM, Yanofsky DJ (2019). Inhibition of mitochondrial translation overcomes venetoclax resistance in AML through activation of the integrated stress response. Science translational medicine.

[10] Tambaro FP, Wierda WG (2020). Tumour lysis syndrome in patients with chronic lymphocytic leukaemia treated with BCL-2 inhibitors: risk factors, prophylaxis, and treatment recommendations. The Lancet Haematology.

[11] Khwaja A, Bjorkholm M, Gale RE, Levine RL, Jordan CT, Ehninger G, Bloomfield CD, Estey E, Burnett A, Cornelissen JJ (2016). Acute myeloid leukaemia. Nat Rev Dis Primers.

[12] Bezerra MF, Lima AS, Piqué-Borràs MR, Silveira DR, Coelho-Silva JL (2020). Co-occurrence of DNMT3A, NPM1, FLT3 mutations identifies a subset of acute myeloid leukemia with adverse prognosis. Blood.

[13] Li J, Liang L, Yang Y, Li X, Ma Y (2021). N-methyladenosine as a biological and clinical determinant in colorectal cancer: progression and future direction. Theranostics.

[14] Gauthier J, Hirayama AV, Purushe J, Hay KA, Lymp J, Li DH (2020). Feasibility and efficacy of CD19-targeted CAR T cells with concurrent ibrutinib for CLL after ibrutinib failure. Blood.

[15] Chen XY, Zhang J, Zhu JS (2019). The role of mA RNA methylation in human cancer. Molecular cancer.

[16] Zhao Q, Zhao Y, Hu W, Zhang Y, Wu X, Lu J, Li M, Li W, Wu W, Wang J (2020). mA RNA modification modulates PI3K/Akt/mTOR signal pathway in Gastrointestinal Cancer. Theranostics.

[17] Huang Y, Su R, Sheng Y, Dong L, Dong Z, Xu H, Ni T (2019). Small-Molecule Targeting of Oncogenic FTO Demethylase in Acute Myeloid Leukemia. Cancer Cell.

[18] Li ZJ, Weng HY, Su R, Weng XC, Zuo ZX, Li CY, Huang HL, Nachtergaele S, Dong L, Hu C (2017). FTO Plays an Oncogenic Role in Acute Myeloid Leukemia as a N-6-Methyladenosine RNA Demethylase. Cancer Cell.

[19] Chen B, Ye F, Yu L, Jia G, Huang X, Zhang X, Peng S, Chen K, Wang M, Gong S (2012). Development of cell-active N6-methyladenosine RNA demethylase FTO inhibitor. J Am Chem Soc.

[20] Huang Y, Yan J, Li Q, Li J, Gong S, Zhou H, Gan J, Jiang H, Jia GF, Luo C (2015). Meclofenamic acid selectively inhibits FTO demethylation of m6A over ALKBH5. Nucleic Acids Res.

[21] Su R, Dong L, Li C, Nachtergaele S, Wunderlich M, Qing Y, Deng X, Wang Y, Weng X, Hu C (2018). R-2HG Exhibits Anti-tumor Activity by Targeting FTO/m(6)A/MYC/CEBPA Signaling. Cell.

[22] Kvasnica M, Urban M, Dickinson NJ, Sarek J (2015). Pentacyclic triterpenoids with nitrogen- and sulfur-containing heterocycles: synthesis and medicinal significance. Nat Prod Rep.

[23] Hill RA, Connolly JD (2020). Triterpenoids. Nat Prod Rep.

[24] Ren M, McGowan E, Li Y, Zhu X, Lu X, Zhu Z, Lin Y, He S (2019). Saikosaponin-d Suppresses COX2 Through p-STAT3/C/EBPbeta Signaling Pathway in Liver Cancer: A Novel Mechanism of Action. Front Pharmacol.

[25] Zhang CY, Jiang ZM, Ma XF, Li Y, Liu XZ, Li LL, Wu WH, Wang T (2019). Saikosaponin-d Inhibits the Hepatoma Cells and Enhances Chemosensitivity Through SENP5-Dependent Inhibition of Gli1 SUMOylation Under Hypoxia. Frontiers in pharmacology.

[26] Wong VKW, Li T, Law BYK, Ma EDL, Yip NC, Michelangeli F, Law CKM, Zhang MM, Lam KYC, Chan PL (2013). Saikosaponin-d, a novel SERCA inhibitor, induces autophagic cell death in apoptosis-defective cells. Cell Death & Disease.

[27] Wang JX, Qi H, Zhang XL, Si W, Xu FF, Hou T, Zhou H, Wang AH, Li GH, Liu YF (2018). Saikosaponin D from Radix Bupleuri suppresses triple-negative breast cancer cell growth by targeting beta-catenin signaling. Biomedicine & Pharmacotherapy.

[28] Deng R, Shen N, Yang Y, Yu H, Xu S, Yang YW, Liu S, Meguellati K, Yan F (2018). Targeting epigenetic pathway with gold nanoparticles for acute myeloid leukemia therapy. Biomaterials.

[29] Yan F, Shen N, Pang JX, Zhao N, Zhang YW, Bode AM, Al-Kali A, Litzow MR, Li B, Liu SJ (2018). A vicious loop of fatty acid-binding protein 4 and DNA methyltransferase 1 promotes acute myeloid leukemia and acts as a therapeutic target. Leukemia.

[30] Yan F, Al-Kali A, Zhang Z, Liu J, Pang J, Zhao N, He C, Litzow MR, Liu S (2018). A dynamic N(6)-methyladenosine methylome regulates intrinsic and acquired resistance to tyrosine kinase inhibitors. Cell Res.

[31] Martinez Molina D, Nordlund P (2016). The Cellular Thermal Shift Assay. A Novel Biophysical Assay for *In situ* Drug Target Engagement and Mechanistic Biomarker Studies. Annu Rev Pharmacol Toxicol.

[32] Zheng GQ, Dahl JA, Niu YM, Fedorcsak P, Huang CM, Li CJ, Vagbo CB, Shi Y, Wang WL, Song SH (2013). ALKBH5 Is a Mammalian RNA Demethylase that Impacts RNA Metabolism and Mouse Fertility. Mol Cell.

[33] Jia GF, Fu Y, Zhao X, Dai Q, Zheng GQ, Yang Y, Yi CQ, Lindahl T, Pan T, Yang YG (2011). N6-Methyladenosine in nuclear RNA is a major substrate of the obesity-associated FTO. Nat Chem Biol.

[34] Sun K, Yu W, Ji B, Chen C, Yang H, Du Y, Song M, Cai H, Yan F, Su R (2020). Saikosaponin D loaded macrophage membrane-biomimetic nanoparticles target angiogenic signaling for breast cancer therapy. Applied Materials Today.

[35] Dou L, Yan F, Pang J, Zheng D, Li D, Gao L, Wang L, Xu Y, Shi J, Wang Q (2019). Protein lysine 43 methylation by EZH1 promotes AML1-ETO transcriptional repression in leukemia. Nat Commun.

[36] Lin X, Chai G, Wu Y, Li J, Chen F, Liu J, Luo G, Tauler J, Du J, Lin S (2019). RNA m(6)A methylation regulates the epithelial mesenchymal transition of cancer cells and translation of Snail. Nat Commun.

[37] Li Z, Weng H, Su R, Weng X, Zuo Z, Li C, Huang H, Nachtergaele S, Dong L, Hu C (2017). FTO Plays an Oncogenic Role in Acute Myeloid Leukemia as a N(6)-Methyladenosine RNA Demethylase. Cancer Cell.

[38] Huang Y, Su R, Sheng Y, Dong L, Dong Z, Xu H, Ni T, Zhang ZS, Zhang T, Li C (2019). Small-Molecule Targeting of Oncogenic FTO Demethylase in Acute Myeloid Leukemia. Cancer Cell.

[39] Deng R, Ji B, Yu H, Bao W, Yang Z, Yu Y, Cui Y, Du Y, Song M, Liu S (2019). Multifunctional Gold Nanoparticles Overcome MicroRNA Regulatory Network Mediated-Multidrug Resistant Leukemia. Sci Rep.

[40] Cui L, Li C, Zhuo Y, Yang L, Cui N, Li Y, Zhang S (2020). Saikosaponin A inhibits the activation of pancreatic stellate cells by suppressing autophagy and the NLRP3 inflammasome via the AMPK/mTOR pathway. Biomed Pharmacother.

[41] Luo H, Vong CT, Chen H, Gao Y, Lyu P, Qiu L Naturally occurring anti-cancer compounds: shining from Chinese herbal medicine. 2019, 14:48.

[42] Kazi J, Rönnstrand L (2019). FMS-like Tyrosine Kinase 3/FLT3. From Basic Science to Clinical Implications. Physiological reviews.

[43] Shen N, Yan F, Pang J, Zhao N, Gangat N, Wu L, Bode AM, Al-Kali A, Litzow MR, Liu S (2017). Inactivation of Receptor Tyrosine Kinases Reverts Aberrant DNA Methylation in Acute Myeloid Leukemia. Clin Cancer Res.

