# Supplementary material for: Saikosaponin D exhibits anti-leukemic activity by targeting FTO/m6A signaling
Source: Theranostics. 2021 Mar 31;11(12):5831–46. doi: 10.7150/thno.55574 (PMC8058711; doi:10.7150/thno.55574)
Supplement: Supplementary file 1 — Supplementary figures. [file thnov11p5831s1.pdf]

Supplementary Materials for

Saikosaponin D exhibits anti-leukemic activity by targeting FTO/m^{6}A signaling

Kaiju Sun[1], Yangyang Du[2], Yuzhu Hou[1], Mingyue Zhao[1], Jiajia Li[3], Yazhe Du[4], Lingxiao Zhang[2], Changbao Chen[1], Hongmei Yang[1], Fei Yan[2,*], Rui Su[1*]

[1]Jilin Ginseng Academy, Changchun University of Chinese Medicine, Changchun, 130017, China

[2]State Key Laboratory of Inorganic Synthesis and Preparative Chemistry, International Joint Research Laboratory of Nano-Micro Architecture Chemistry (NMAC), International Research Center for Chemistry-Medicine Joint Innovation, College of Chemistry, Jilin University, 2699 Qianjin Street, Changchun 130012, China

[3]Department of Obstetrics and Gynecology, First Hospital, Jilin University, 130021, P. R. China

[4]Department of blood specialty, First Hospital, Jilin University, 130021, China

[*]Correspondence and requests for materials should be addressed to F. Y(feiyan@jlu.edu.cn) or R. S. (sranalchem@163.com).

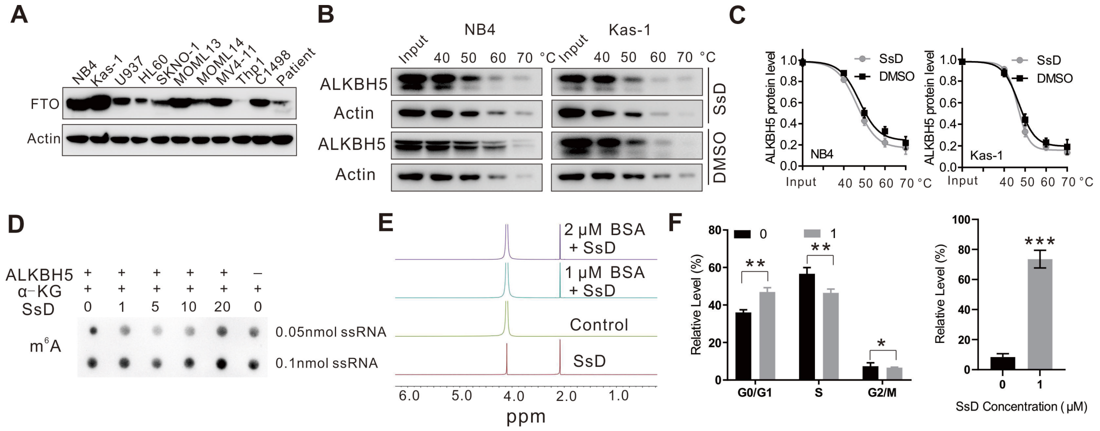

Figure S1. (A) Quantification of SsD on cell-cycle arrest and apoptosis using FACS based on PI staining in NB4 cells after 48 h of treatment. Data are mean ± SD; *P < 0.05, **P < 0.01， ***P < 0.001. (B) The expression analysis of FTO in AML cells. (C-D) Western blot analysis determining the effects of 1 μM SsD on thermal stabilization of ALKBH5 protein is shown. CETSA assay was performed in cell lysates. The results were derived from three biological replicates. (E) Dot blot analysis of m^6A abundance in the presence of various SsD concentrations, α-KG and ALKBH5 protein in a cell-free system. (F) NMR measurement of SsD interaction with BSA.

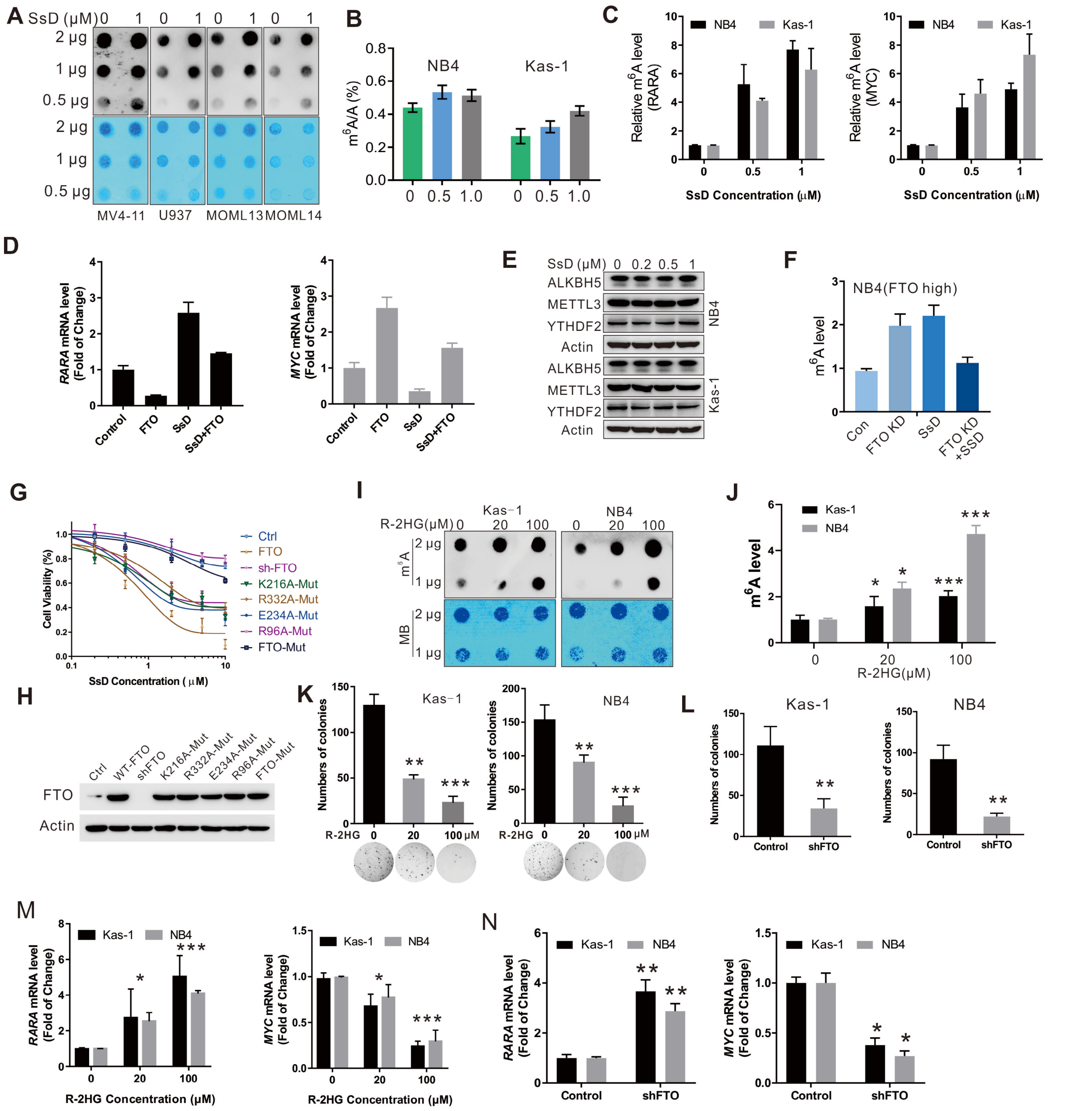

Figure S2. (A) m^{6}A dot blot analysis of the indicated cells treated with SsD. MB (methylene blue) represents the loading control of RNA samples. (B) Quantitation of m^{6}A/A ratios in mRNA using LC-MS/MS in NB4 and Kas-1 cells treated with SsD for 48 h is shown. (C) Gene-specific m^{6}A qPCR of indicated genes in SsD treated NB4 or Kas-1 cells. (D) NB4 cells were treated with 0.5 μM of SsD for 24 h followed by FTO forced expression plasmid transfection for additional 24 h. The cells transfected with FTO forced expression plasmid or SsD alone as compared. (E) Western blotting protein level analysis of the indicated genes in SsD treated cells. (F)

Quantitation of m^6A levels in the indicated groups using dot blotting is shown. (G) CCK-8 assays for THP-1 cells transected with indicated plasmid or shRNA for 24h followed by SsD treatment for additional 48h. (H) The expression analysis of FTO in indicated transfected cells. (I) m^6A dot blot analysis of the indicated cells treated withR-2HG. MB (methylene blue) represents the loading control of RNA samples. (J) Quantification of m^6A dot blot analysis of the indicated cells treated with R-2HG. (K and L) Colony-forming assays in NB4, Kas-1, treated with different concentrations of R-2HG (K) or FTO shRNA (L) and Graphs showing the colonies numbers from 3 independent experiments. (M, N) qPCR expression analysis of indicated genes in R-2HG (M) or FTO shRNA (N) treated cells. Data represent three independent experiments.

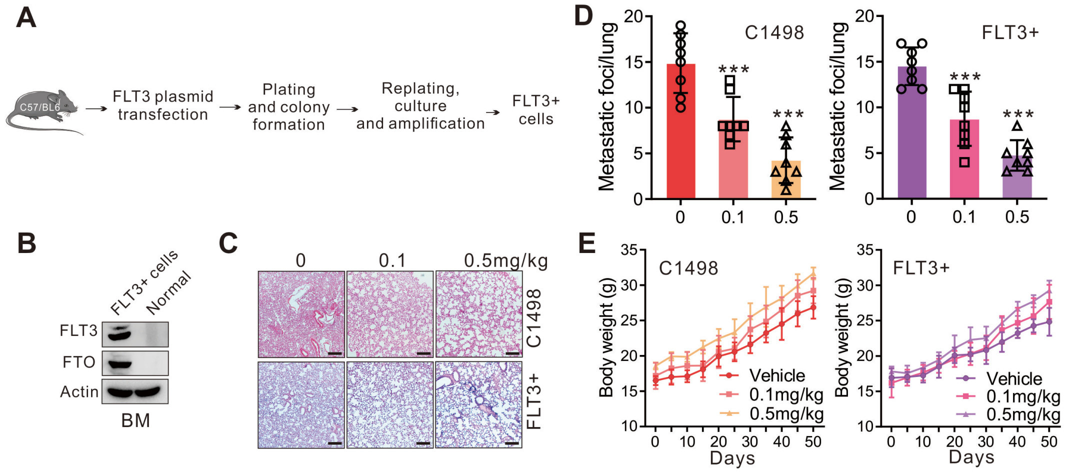

Figure S3. (A) Establishment of FLT3 + cell model. (B) Western blot analysis of the FTO protein level in FLT3+ and normal mouse bone marrow cells. (C) Pictures show representative external views of H&E-stained lung sections (Scale bars, 1 cm). (D) The quantification of tumor nodules growing on the lung is shown. (E) Change in the bodyweight of all animal groups during the 50 days of the study is shown.

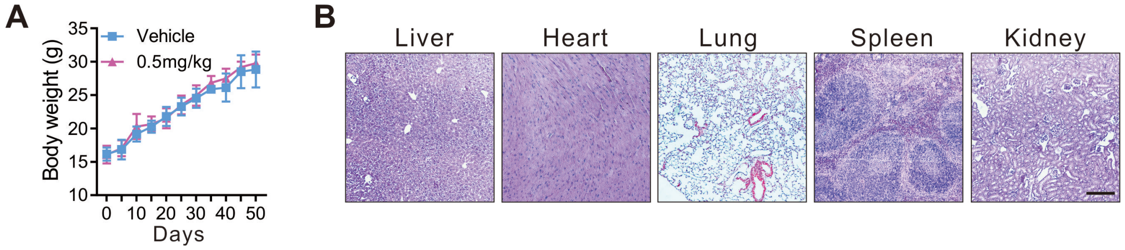

Figure S4. (A) Bodyweight of normal mice treated with PBS or SsD (n=3) is shown. (B) Images are showing H&E-stained tissue sections of normal mice treated with SsD (0.5 mg/kg).

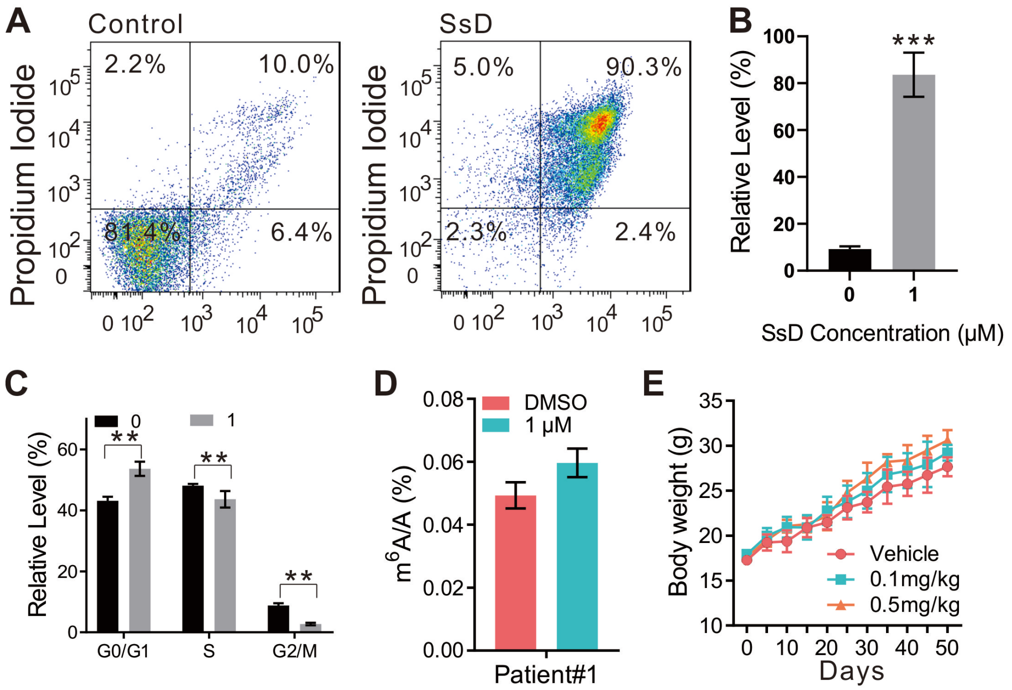

Figure S5. (A) Effect on apoptosis in patient cells after 48 h of SsD treatment was determined using FACS based on PI staining. (B-C) Quantification of SsD on cell-cycle arrest and apoptosis using FACS based on PI staining in patient cells after 48 h of treatment. Data are mean ± SD; *P < 0.05, **P < 0.01， ***P < 0.001. (D) Quantitation of the m^6A/A ratios in mRNA using LC-MS/MS in patient cells treated with 1 μM for 48 h is shown. (E) Change in the bodyweight of all animal groups during 50 days is shown.

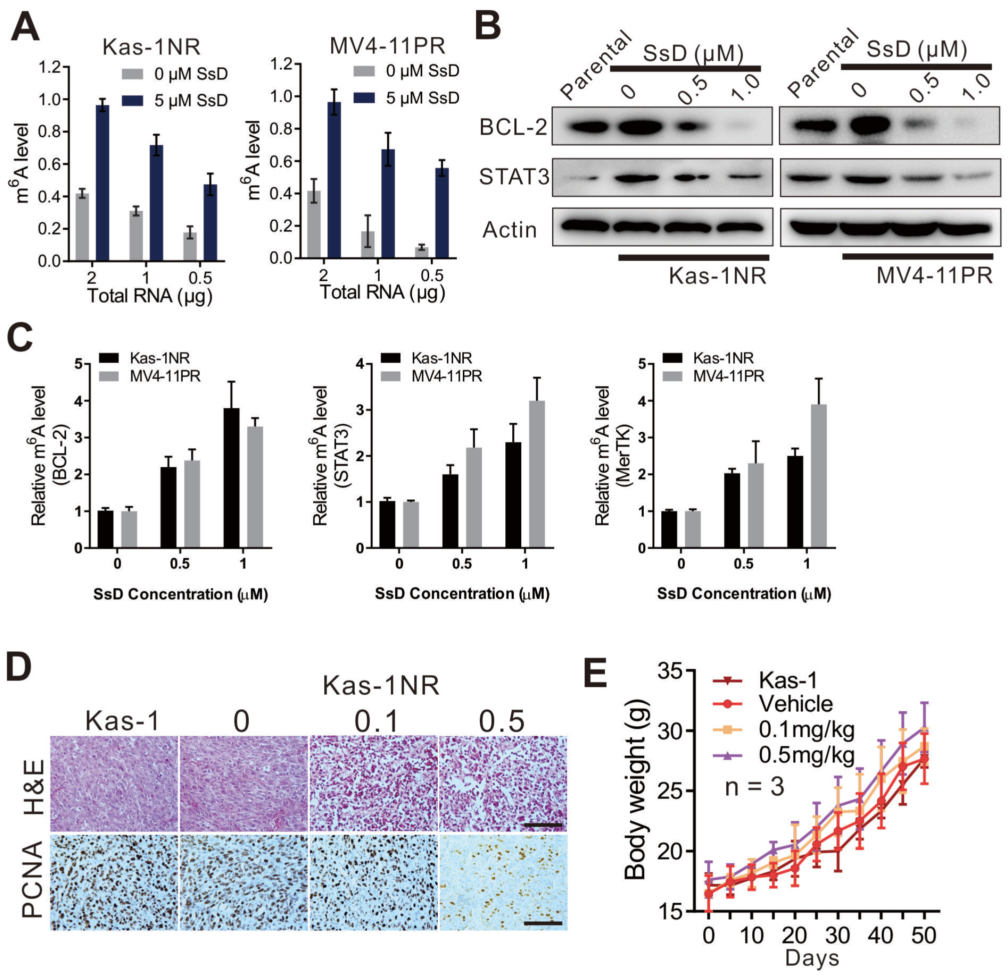

Figure S6. (A) Quantitative dot blot analysis of the m^{6}A from the indicated cells treated with SsD. (B) Western blot protein level analysis of Kas-1NR and MV4-11PR cells treated with SsD. (C) Gene-specific m^{6}A qPCR of indicated genes in SsD treated TKI resistant cells. (D) Images for PCNA staining of tumor sections from tumor-bearing mice are shown. Scale bar =100 μm. (E) Change in the bodyweight of all animal groups during 50 days of study is shown.

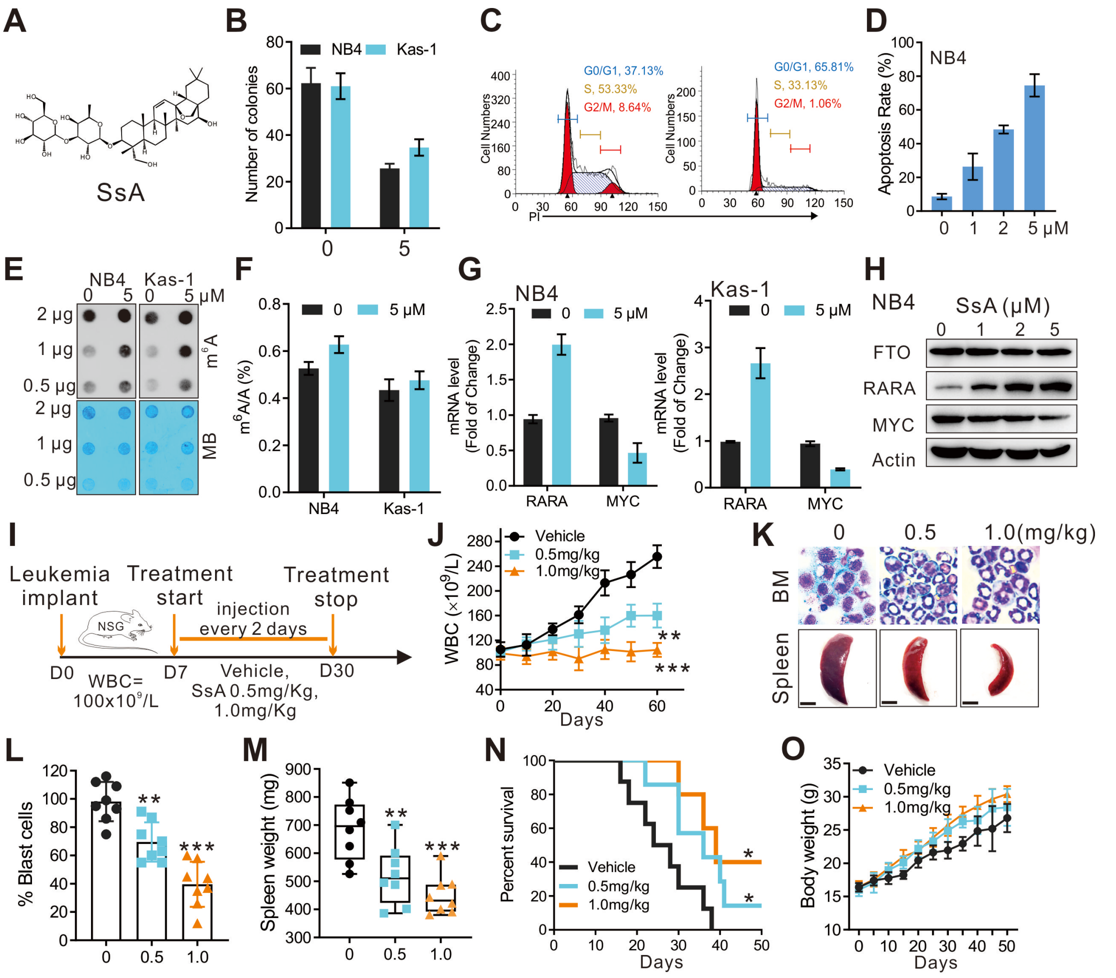

Figure S7. (A) The molecular structure of SsA is shown. (B) The colony numbers in SsA treated NB4 and Kasumi-1 cells are presented. (C-D) Determination of the effect of SsA on cell-cycle arrest (C) and apoptosis (D) using FACS based on PI staining in NB4 cells after 48 h of SsA treatment. (E) Dot blot analysis of m^{6}A from indicated cells treated with SsA is shown. MB (methylene blue) represents the loading control of RNA samples. (F) Quantitation of m^{6}A/A ratios in mRNA using LC-MS/MS in NB4 cells treated with 5 μM SsA for 48 h. (G) qPCR expression analysis of indicated genes in SsA treated cells. Data represent three independent experiments. (H) Western blot protein level analysis of indicated genes in SsA treated cells. (I) Leukemia-bearing mice were developed by the intravenous injection of C1498 cells

into 4 weeks old C57BL/6 mice. (J) WBC count of leukemia-bearing mice (n = 8) is shown. (K) Pictures show representative external views of the spleen (Scale bars, 1 cm, lower) and Wright-Giemsa-stained BM cells (upper). (L-M) Quantification of blast cells and spleen weight is shown. (N) The survival curve of leukemia-bearing mice was calculated using the Kaplan-Meier estimate (n=8). (O) Change in the bodyweight of all animal groups during 50 days is shown.
